# *Sanguisorba officinalis* L. Suppresses Triple-Negative Breast Cancer Metastasis by Inhibiting Late-Phase Autophagy via Hif-1α/Caveolin-1 Signaling

**DOI:** 10.3389/fphar.2020.591400

**Published:** 2020-12-14

**Authors:** Neng Wang, Gulizeba Muhetaer, Xiaotong Zhang, Bowen Yang, Caiwei Wang, Yu Zhang, Xuan Wang, Juping Zhang, Shengqi Wang, Yifeng Zheng, Fengxue Zhang, Zhiyu Wang

**Affiliations:** ^1^ The Research Center for Integrative Medicine, School of Basic Medical Sciences, Guangzhou University of Chinese Medicine, Guangdong, China; ^2^ Integrative Research Laboratory of Breast Cancer, The Second Clinical College, Guangzhou University of Chinese Medicine, Guangdong, China; ^3^ Guangdong Provincial Key Laboratory of Clinical Research on Traditional Chinese Medicine Syndrome, Guangdong Provincial Academy of Chinese Medical Sciences, Guangdong Provincial Hospital of Chinese Medicine, Guangdong, China

**Keywords:** breast-cancer metastasis, *Sanguisorba officinalis* L., late-phase autophagic regulation, Cav-1, Hif-1α

## Abstract

*Sanguisorba officinalis* L. (SA) is a common herb for cancer treatment in the clinic, particularly during the consolidation phase to prevent occurrence or metastasis. Nevertheless, there are limited studies reporting the molecular mechanisms about its anti-metastatic function. It is well demonstrated that autophagy is one of the critical mechanisms accounting for metastasis and anti-cancer pharmacological actions of Chinese herbs. On the threshold, the regulatory effects and molecular mechanisms of SA in suppressing autophagy-related breast cancer metastasis were investigated in this study. *In vitro* findings demonstrated that SA potently suppressed the proliferation, colony formations well as metastasis process in triple-negative breast cancer. Network and biological analyses predicted that SA mainly targeted caveolin-1 (Cav-1) to induce anti-metastatic effects, and one of the core mechanisms was *via* regulation of autophagy. Further experiments—including western blotting, transmission electron microscopy, GFP-mRFP-LC3 immunofluorescence, and lysosomal-activity detection—validated SA as a potent late-stage autophagic inhibitor by increasing microtubule-associated light chain 3-II (LC3-II) conversion, decreasing acidic vesicular-organelle formation, and inducing lysosomal dysfunction even under conditions of either starvation or hypoxia. Furthermore, the anti-autophagic and anti-metastatic activity of SA was Cav-1-dependent. Specifically, Cav-1 knockdown significantly facilitated SA-mediated inhibition of autophagy and metastasis. Furthermore, hypoxia inducible factor-1α (Hif-1α) overexpression attenuated the SA-induced inhibitory activities on Cav-1, autophagy, and metastasis, indicating that SA may have inhibited autophagy-related metastasis *via* Hif-1α/Cav-1 signaling. In both mouse breast cancer xenograft and zebrafish xenotransplantation models, SA inhibited breast cancer growth and inhibited late-phase autophagy *in vivo*, which was accompanied by suppression of Hif-1α/Cav-1 signaling and the epithelial-mesenchymal transition. Overall, our findings not only indicate that SA acts as a novel late-phase autophagic inhibitor with anti-metastatic activities in triple-negative breast cancer, but also highlight Cav-1 as a regulator in controlling late-phase autophagic activity.

## Introduction

Breast cancer undoubtedly ranks the most frequent female malignancy and the second-leading fatal reason for women, with 268,600 new cases and 41,760 related deaths among female populations in the United States in 2019 (Siegel et al., 2019; Britt et al., 2020). Although remarkable endeavor has been made for seeking therapeutic strategies and combating breast cancer, metastasis is still a major clinical challenge that accounts for the primary cause of cancer-related mortalities, particularly for triple-negative breast cancer. Breast cancer exhibits metastatic heterogeneity to distinct organs—including bones, lungs, the liver, and the brain—which has led to resistance to various standard treatments in clinical trials (Kimbung et al., 2015). The five-year overall survival (OS) rate has been considered to be as high as 98.8% among patients diagnosed with localized breast tumors. Nevertheless, 90% of cancer-related deaths are attributable to metastasis, resulting in a dramatic reduction in the reported OS to approximately 26.3% among women with distant metastatic lesions (Peto et al., 2012; Wang et al., 2017). In addition, 10–20.8% of breast cancer cases are initially diagnosed as triple-negative phenotypes, while 20–50% of localized primary cases eventually develop into distant recurrent phenotypes even following successful primary tumor resection and adjuvant therapy (Lu et al., 2009). As such, the suppression of metastasis is an expectable therapeutic target of breast cancer, and deciphering the involved metastatic mechanisms may speed up improving effective strategies against breast cancer.

Autophagy refers to the engulfment of cytoplasmic cargo into autophagosomes characterized by double membranes, in order to subsequently delivering the transported cargo into lysosomes for degradation mediated by a series of acidic hydrolase enzymes (Dower et al., 2018). It has been reported that more than 30 autophagy-related (ATG) genes in the regulatory process of autophagy, which consists of five general steps including autophagosomal biogenesis, phagophore nucleation and expansion, autophagosome maturation, autophagosome fusion with lysosome, as well as degradation of the engulfed materials (Mizushima, 2007). The regulation of autophagy has now been regarded as being implicated in various cancers. Lai et al. (2020) identified and analyzed four ATGs (ATG4A, IFNG, NRG1, and SERPINA1) associated with poor OS in breast cancer patients, as determined *via* comprehensive analysis of ATGs derived from The Cancer Genome Atlas (TCGA) database. Dower et al. (2018) further revealed multiple autophagy-based regulations during tumoral metastasis, including focal adhesion, anoikis resistance, tumor-stroma interaction, epithelial-mesenchymal transition (EMT), fibrosis as well as Rho GTPase activity. In addition, the role of autophagy in cancer metastasis is multifaceted due to different cell types and numerous extracellular and intracellular stressors derived from tumor microenvironment (TME) (Maes et al., 2013). Most frequently, autophagy prevents cancer cells from being harmed by severe stimuli such as nutrient deprivation, hypoxia, oxidative stress, unfolded/misfolded proteins, irradiation, heat sock, and microbial invasion (Kubisch et al., 2013; Daskalaki et al., 2018). Among these stimuli, nutrient deprivation and hypoxia represent common features of the TME. Under starvation, autophagy is activated to meet the bioenergetic demands of cells by lysosomal recycling and reutilization of metabolites. It was found that autophagy catabolically started an operation for oxidative repair by eliminating mitochondriae with damage or excessive reactive oxygen species (ROS) (Morselli et al., 2009). With regard to hypoxic stress, Hif-1α has been extensively explored in several studies regarding hypoxia-induced autophagy during cancer metastasis (Daskalaki et al., 2018). For instance, Zhang et al. (2018) reported that LncRNA CPS1-IT1 blocked hypoxia-dependent autophagy and subsequently suppressed the colorectal cancer metastasis mainly by inactivation of the target Hif-1α. Wang et al. identified beclin-1 co-expression with Hif-1α as a predictor for early distant metastasis and dropped survival rates in breast cancer patients with ER-positive and HER2-negative phenotypes (Dong et al., 2013). Currently, multiple anti-autophagy compounds are discovered, but only chloroquine (CQ) and hydroxychloroquine (HCQ) have been approved for clinic application (Long and McWilliams, 2019; Wang et al., 2019). To date, HCQ has been tested as a novel anti-cancer agent for different phases of clinical trials, but its inability in minimizing off-target toxicity and regulating autophagic activity largely restricted its therapeutic window (Limpert et al., 2018). Therefore, it is urgent to looking for more advanced and much safer inhibitors targeting autophagy from natural sources.

Traditional Chinese medicine (TCM) has been reputable for its long-term application as a complementary therapeutic strategy against cancers in China and throughout the world. Specifically, TCM has been widely used as a pool filed with abundant novel therapeutic agents for approximately 63–83% of patients with breast cancer, owing to its favorable efficacies, accessible availability, and limited adverse effects (Liu et al., 2019; Yang et al., 2019). *Sanguisorba officinalis* L (SA, Chinese name as Di Yu; The voucher number/barcode 2,896,446) is traditionally prescribed for cancer prevention and treatment that putatively works by reducing blood toxicity and enhancing immunity in patients (Bai et al., 2019). In our early study, we found SA exerted an obvious inhibitory effect on breast cancer angiogenesis, which is known to represent the basis of tumor growth, invasion, and metastasis (Wang et al., 2012b). In a more recent study, it was further demonstrated that one of the components of SA, namely ellagic acid, limited breast cancer stem cell metastasis by directly targeting ACTN4 and subsequently promoting *β*-catenin destabilization (Wang et al., 2017). However, the roles of SA and its active compounds on autophagic regulation have remained unclear since autophagic flux was not explicitly examined in earlier studies. Importantly, it brings profound significance from the patient’s bedside to the laboratory bench by evaluating the involved pharmacology and toxicology of certain herbal medicines with good clinical efficacy (Wang et al., 2008). Given that SA has always been demonstrated with favorable clinical efficacy against breast cancer, it is worthwhile to further investigate its role in autophagy-dependent metastasis and to identify the precise molecular targets that are involved.

In the present research, we discovered that SA restrained growth and metastasis by suppressing late-phase autophagy in triple-negative breast carcinoma. Furthermore, a drug-target-disease network was established and indicated that SA mainly targeted Cav-1 to induce anti-metastatic effects. We further demonstrated that SA inhibited breast cancer metastasis primarily by restraining late-stage autophagy *via* Hif-1α/Cav-1 signaling. Taken together, our findings not only imply the SA application for the treatment of advanced breast cancer, but also highlight a diagnostic performance of Cav-1 in the regulation of tumoral autophagy.

## Materials and Methods

### The Preparation of *Sanguisorba Officinalis* L.

The preparation and standardization of SA (Guangdong Provincial Hospital of Chinese Medicine, Guangzhou, China) were conducted following the protocol reported in our pilot study (Wang et al., 2012b). For drug preparation, SA underwent mechanical trituration into a fine powder (200 g), efflux extraction for 1 h thrice with 2 L water, inspissation, and lyophilization to a final production ratio of 9.6%–12.4%. Quality control analysis was achieved with an Agilent 1200 system (Agilent, CA, USA) combined with diode array detection (DAD) and an Agilent C18 column (5 μm, 250 mm × 4.6 mm) with a high-performance liquid chromatography (HPLC) guard cartridge system (Phenomenex, CA, USA). The mobile phases consisted of acetonitrile with 0.1% (v/v) formic acid (A) and water with 0.1% (v/v) formic acid (B) using a gradient program of 5–15% A for 0–10 min, 15–25% A for 10–25 min, 25–30% A for 25–35 min, 30–70% A for 35–45 min, 70–95% A for 45–50 min, and 95% A in 50–60 min. The flow rate was 1.0 ml/min, the column temperature was set to 35°C, and the detection wavelength was set at 272 nm. The standard solutions of gallic acid (GA) were prepared and diluted with methanol for linearity studies. Specifically, 10 μL of the SA solutions were injected for HPLC analysis and a calibration curve was constructed by plotting the peak areas against the concentrations of the analytes. For assaying of the contents of GA of SA, 0.1 g of SA was weighed in a 100-ml Erlenmeyer flask and was added to 10 ml of water. Then, the solution was sonicated for 60 min, normalized to 10 ml by adding additional water, filtrated through a 0.2-μm membrane filter, and lastly injected for HPLC analysis. The concentration of GA in SA samples was controlled between 1.78% and 2.15%.

### Cell Culture

Human breast cancer cell lines MDA-MB-231, BT-549, and MCF-7 were derived from the American Type Culture Collection. The non-malignant mammary epithelial cell line, HBL-100, was purchased from the National Infrastructure of Cell Line Resource. Dulbecco’s modified eagle medium (DMEM, Gibco) for MDA-MB-231, BT-549, and HBL-100 cells and RPMI-1640 medium (Gibco) for MCF-7 cell were employed in addition to 10% fetal bovine serum (Gibco) and 1% penicillin and streptomycin (Gibco) at 37°C with 5% CO_2._

### Cell Viability Assay and Colony Formation Assay

For CCK-8 assays, cells were seeded at a concentration of 3 × 10^3^ cells per well in 96-well plates overnight. The effects of SA on cell viability were analyzed with CCK-8 kits (KeyGEN BioTECH, Nanjing, China). For cell counting analysis, cells were planted into 6-well plates at a concentration of 0.5 × 10^6^ cells per well overnight. Cells were counted with trypan-blue exclusion on a Cellometer Mini device (Nexcelom, Boston, USA) after the indicated treatment. For colony formation detection, cells were cultured in 6-well plates at a density of 1 × 10^3^ cells per well overnight. The attached cells were exposed to SA for 4  h, and then cultivated with fresh complete culture medium for additional two weeks. The formed colonies were fixed with 4% paraformaldehyde, dyed with Coomassie blue, and then counted for examination.

### Detection of Apoptosis

For flow cytometry analysis, cells were seeded onto 6-well plates at a density of 3 × 10^5^ cells per well overnight. SA-treated cells were dyed with Annexin V-FITC/PI apoptosis detection kit (KeyGEN BioTECH, Nanjing, China), and analyzed by the system of FACSAria flow cytometer (BD Biosciences, New Jersey, USA), and FlowJo software. For Hoechst 33,258 staining, cells were seeded in 6-well plates at 60–70% confluency, treated with SA, and stained with Hoechst 33,258 dye for 10 min at room temperature before examination (KeyGEN BioTECH, Nanjing, China).

### Wound Healing Assay and Transwell Invasive Assay

In regard to wound healing assays, 4 × 10^5^ cells were seeded onto 6-well plates for over 90% confluency and scratched with a 1-ml pipette tip. Next, cells were exposed to the indicated treatment, washed thrice with PBS, and recorded at 0, 24, and 48 h after initiation of the wound healing. The healing distance and healing area were analyzed by Image J software (National Institute of Mental Health, Bethesda, Maryland, USA). For transwell invasive assays, 8-μm transwell chambers (Corning, New York, USA) were coated with Matrigel (Corning, New York, USA) and placed into 24-well plates before experiment. Subsequently, the upper chambers were added with cells at a density of 1.5 × 10^5^ following the indicated treatment, whereas the lower chamber was filled with complete DMEM medium containing 10% FBS for 24 h. Finally, the upper cells were erased with a cotton swab, while the invaded cells into the bottom of chamber were fixed with 4% formaldehyde, dyed with 0.5% hematoxylin solution for 20 min, and photographed for examination.

### Western Blotting

Cells or tissues were lysed with RIPA buffer (Sigma, St. Louis, MO) containing protease inhibitors (Roche Diagnostics, IN), quantified by the bicinchoninic-acid assay kit (Thermo Fisher Scientific, Bonn, Germany). Protein lysates (30 µg) were separated by 10% or 12% sodium-dodecyl-sulfate polyacrylamide gel electrophoresis (SDS-PAGE), and transferred onto a PVDF membrane (Millipore, Billerica, MA, USA). Blocked membranes were incubated with the primary antibodies including E-cadherin (E-cad, no. 20874-1-AP), N-cadherin (N-cad, no. 66219-1-AP), vimentin (no. 60330-1-AP), Cav-1 (no.16447-1-AP), LC3 (no. 14600-1-AP), and p62/SQSTM1 (no. 18420-1-AP) from Proteintech (Rosemont, USA); Hif-1α (no. 3716S) and *β*-actin (no. 3700S) from CST (Boston, MA, USA), and the corresponding secondary anti-rabbit or anti-mouse (Proteintech, Rosemont, USA), followed by visualization with chemiluminescence detection (Tanon, Shanghai, China) and quantitative analysis with ImageJ software.

### Matrix Metalloproteinase Zymography Assay

MDA-MB-231 and BT-549 cells were grown in DMEM to confluence, and then exposed to SA in serum-free DMEM for the indicated treatment. Supernatants were then harvested to detect matrix metalloproteinase (MMP) activities by the Zymography Assay Kit (XF-P17750, Xinfan BioTECH, Shanghai, China). The general steps included preparation of SDS-PAGE Gel Containing MMP Substrates, mixture of samples with 2× SDS-PAGE non-reducing buffer (4% SDS, 100 mM Tris-Cl pH6.8, 20% glycerol, 0.02% bromophnol blue), electrophoresis at 20 mA per gel at 4°C, wash of gel with 1 × buffer A for 24 h twice at room temperature, incubation of gel with 1 × buffer B for 5 h at 37°C, and scanning of dyed gel after incubation with Coomassie blue. The location and size of the transparent bands indicated the activities of MMPs.

### Establishment of Herb-Chemical-Target Interaction

The chemical information of SA were collected from Traditional Chinese Medicine Systems Pharmacology (TCMSP) database (http://lsp.nwsuaf.edu.cn/tcmsp.php) based on the criteria of oral bioavailability (OB) ≥ 30% as well as drug likeness (DL) ≥ 0.1. In addition, ellagic acid and gallic acid were also considered as potential candidates due to the bioactivity-guided identification of SA in our pilot study (Wang et al., 2012b). The interaction between chemicals and targets of SA was established with Cytoscape software (Version 3.2.1), and the structure of representative chemicals in SA was drawn with ChemDraw 14.0 Software (CambridgeSoft Corporation, USA).

### Gene Ontology and Pathway Enrichment Analysis

Targets associated with breast cancer were retrieved from dataset GSE 65194 on the platform of Affymetrix GeneChip Human Genome U133 Plus 2.0 Array in the National Center for Biotechnology Information (NCBI) Gene Expression Omnibus (GEO) database. The differentially expressed genes (DEGs) were obtained on the basis of *p* ≤ 0.05 and a fold control (FC) ≥ 1.5 when comparing 130 invasive breast tumors with 11 normal mammary tissues in GSE 65194, and were then delivered to STRING database to evaluate the protein–protein interaction (PPI) information. In addition, the aforementioned DEGs were submitted to the database for Annotation, Visualization, and Integrated Discovery (DAVID; http://david.abcc.ncifcrf.gov/) for the enrichment analysis of gene ontology (GO) and Kyoto Encyclopedia of Genes and Genomes (KEGG).

### Electron Microscopy

Treated cells were harvested and fixed with glutaraldehyde solution (Leagene, Beijing, China) for 1 h at room temperature. The pellets were then exposed to 1% osmium tetroxide/1.5% potassium ferrocyanide, followed by 1% uranyl acetate, dehydrated in graded series of ethanol, and embedded in epon-araldite. Examination of autophagosomes and/or autolysosomes was performed with PhilipCM20 transmission electron microscope.

### Measurement of Autophagic Flux

For monitoring autophagic flux, cells were seeded at a concentration of 2.5 × 10^5^ cells per well in laser-scanning-confocal Petri dishes for 24 h, and then transfected with mRFP-GFP-LC3 adenoviral vectors (HanBio Technology, Shanghai, China) or Lysored (KeyGEN BioTECH, Nanjing, China) or DQ Green BSA (Thermo Fisher Scientific, Waltham, USA) followed by the indicated treatment and examination under a LMS710 confocal microscope (ZEISA).

### Plasmid Transfection and RNA Interference

*pENTER*-HIF-1A plasmid or scrambled plasmid were brought from Vigene Biosciences (Jinan, China), and trasfected intio MDA-MB-231 cells using LipoFiterTM reagent (Hanbio Biotechnology Co., LTD. Shanghai, China). After 48 h transfection, cells were further screened by adding puromycin (Invitrogen, Carlsbad, CA, USA) at 10 μg/ml and passaged for additional two weeks. For small interfering RNA (siRNA) transfection, CAV-1, HIF-1A and control siRNAs were synthesized and purified by Genepharm (Shanghai, China). The mixture of siRNA (5′-GCA​ACA​UCU​ACA​AGC​CCA​ATT-3′) targeting CAV-1 or the siRNA (5′-CCA​CCA​CUG​AUG​AAU​UAA​ATT-3′) targeting HIF-1A with X-tremegene siRNA transfection reagent (Roche Diagnostics, Shanghai, China) were added into cells following the manufacturer’s instructions. After 48 h of transfection of plasmids or siRNAs, cells were used for further studies.

### Breast Cancer Xenotransplantation Model

For a mouse xenotransplantation model, four-week-old female nude mice were provided by Guangdong Medical Animal Experimental Center, and all operations were approved by the Animal Care and Use Committee of Guangzhou University of Chinese Medicine. Particularly, MDA-MB-231 cells at a density of 1 × 10^6^ were mixed with Matrigel (Corning, New York, USA), and implanted into the mammary glands of mice. SA was given at 1 g/kg/day per day by oral gavage when the tumor sizes achieved approximately 5 × 5 mm. To monitor the tumor growth, treated mice were intraperitoneally injected with D-Luciferin (150 mg/kg, PerkinElmer, MA, USA) and photographed with the IVIS imaging system (IVIS-spectrum, Perkin Elmer, Waltham, MA, USA). For the tail vein injection experiments, MDA-MB-231 cells at a density of 4 × 10^5^ were intraperitoneally injected into tail veins of mice, following with SA treatment. Finally, the mice were euthanized and their lungs were removed and compared.

For a zebrafish xenotransplantation model, MDA-MB-231 cells were labeled with 1, 1′-Dioctadecyl-3, 3, 3′, 3′-tetramethylindocarbocyanine perchlorate (DiI, Sigma-Aldrich) at 5 μM in advance or transfected with mRFP-GFP-LC3 adenoviral vectors. 200 labeled cells were suspended in 20 nL DMEM medium for the perivitelline space (PVS)-located injection of each zebrafish embryo after 48 h fertilization, followed by a 48 h treatment of SA diluted with 2 ml of aquaculture water in 48-well plates (one fish per well). Tumor formation or autophagic activation of zebrafish were observed under fluorescent microscopy (Nikon Eclipse C1, Tokyo, Japan) and was quantified *via* ImageJ software.

### Hematoxylin-Eosin Staining and Immunohistochemistry

Paraffin-embedded sectioned were treated with xylene twice 10 min each and rehydrated with gradual ethanol ranging from 100% to 70% and finally immersed into distilled water. For H&E staining, cellular nucleus was visualized with 10% hematoxylin, and cytoplasm was stained with 1% eosin. Next, the slides were dehydrated, cleared, and mounted before pathological observation. For Immunohistochemistry (IHC) analysis, the slides were permeabilized with 0.3% hydrogen peroxide, followed with antigen retrieval by heating specimens in sodium-citrate buffer. After blocking with 10% goat serum, the samples were treated with a primary antibody (1:50 dilution) including Ki67 (no. AF0198, Affinity), Cav-1 (no. 16447-1-AP, Proteintech), Hif-1α (no. 3716S, CST), vimentin (no. 60330-1-AP, Proteintech), and E-cad (no. 20874-1-AP, Proteintech) at 4°C for 24 h, and incubated with the corresponding secondary antibody for 30 min. The sections were further incubated with 3, 3′-diaminobenzidine (DAB) (ZSGB-BIO, Beijing, China) and counterstained with 10% hematoxylin.

### Statistical Analysis

The results were presented as the mean ± standard deviation (SD). Statistical significance was achieved as a *p* ≤ 0.05 with student’s t-tests and one-way analyses of variance (ANOVA). All calculations were performed by SPSS 17.0 software (Abbott Laboratories, Chicago, IL).

## Results

### *Sanguisorba Officinalis* L. Exerts Anti-Proliferation and Apoptosis-Promoting Effects in Breast Cancer Cells

**FIGURE 1:**
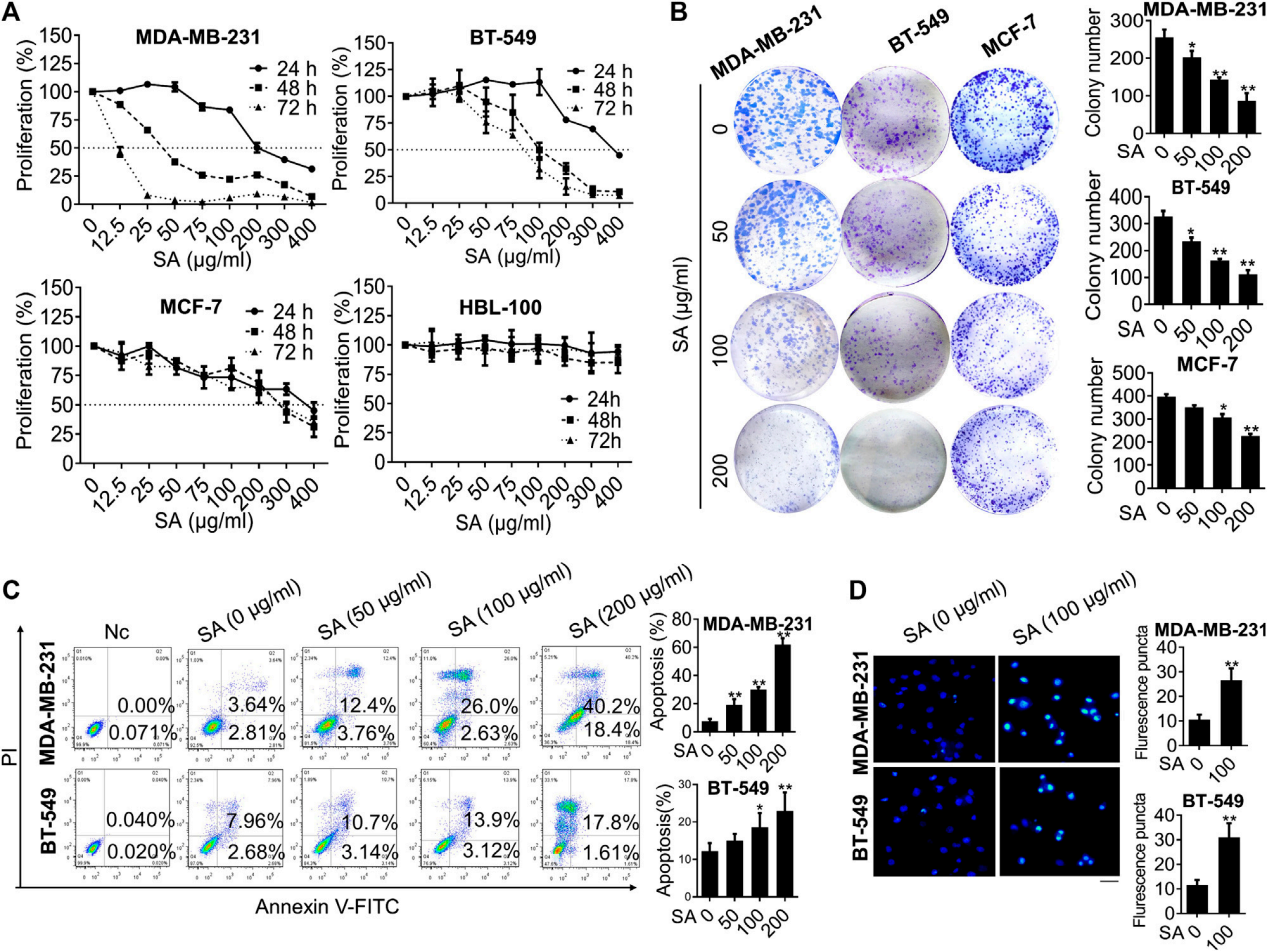
SA exerts anti-proliferation and apoptosis-promoting effects in breast cancer cells. **(A)** Proliferation of the indicated cells treated with either certain concentrations (0–400 μg/ml) of SA for 48 h or certain time intervals (0, 24, and 48 h) of SA by CCK8 assays. **(B)** The influence of SA (0–200 μg/ml) on colony formation of MDA-MB-231, BT-549, and MCF-7 cells. **(C)** The apoptotic populations after a 48-h treatment of SA (0–200 μg/ml) in MDA-MB-231 and BT-549 cells. **(D)** The fluorescence photographs of SA-treated cells stained with Hoechst 33,258 dye (100 μg/ml). Scale bar: 50 μM. All values represent the mean ± SD (*n* = 3, *p < 0.05, **p < 0.01).

After drug preparation and quality control of SA (Supplementary Figure S1), we investigate whether SA could affect the growth of multiple breast cancer cell lines including two triple-negative phenotypes MDA-MB-231 and BT-549, a luminal-like phenotype MCF-7, as well as a non-malignant mammary epithelial cell HBL-100. As shown in Figure 1A, SA dose- and time-dependently suppressed the proliferation of breast cancer cell, with a median inhibitory concentration (IC_50_) of 34.91 μg/ml for MDA-MB-231, 103.74 μg/ml for BT-549, and 215.8 μg/ml for MCF-7 within 48 h. Particularly, SA treatment continued to exert inhibitory effects on MDA-MB-231 and BT-549 cells with high-metastatic potential over the next 72 h, while posed obscure effects on the growth of HBL-100 cells from 24 to 72 h after the indicated incubation. This result was consisted with the investigation of colony formation, in which SA attenuated the growth rate of breast cancer cells ranging from 50 to 200 μg/ml (Figure 1B). Next, flow cytometry analysis was conducted to investigate whether apoptosis induction was responsible for the anti-proliferation activity of SA. Compared with that of untreated cells, a 48-h administration with SA significantly led to increased number of both early and late apoptotic cells (Figure 1C). In addition, Hoechst 33,258 staining was also employed for morphological observation of the apoptotic cells by fluorescent imaging, as previously described (Zhang et al., 2010). As confirmed by Hoechst 33,258 staining, it was found that the SA-treated breast cancer cells presented typical characteristic behavior of apoptosis such as brighter chromatin staining and cell pyknosis (Figure 1D). Taken together, the data indicated that SA could suppress triple-negative breast cancer cell growth and promoted apoptosis *in vitro*.

### *Sanguisorba Officinalis* L. Attenuates Migrative and Invasive Potential of Metastatic Cells

**FIGURE 2:**
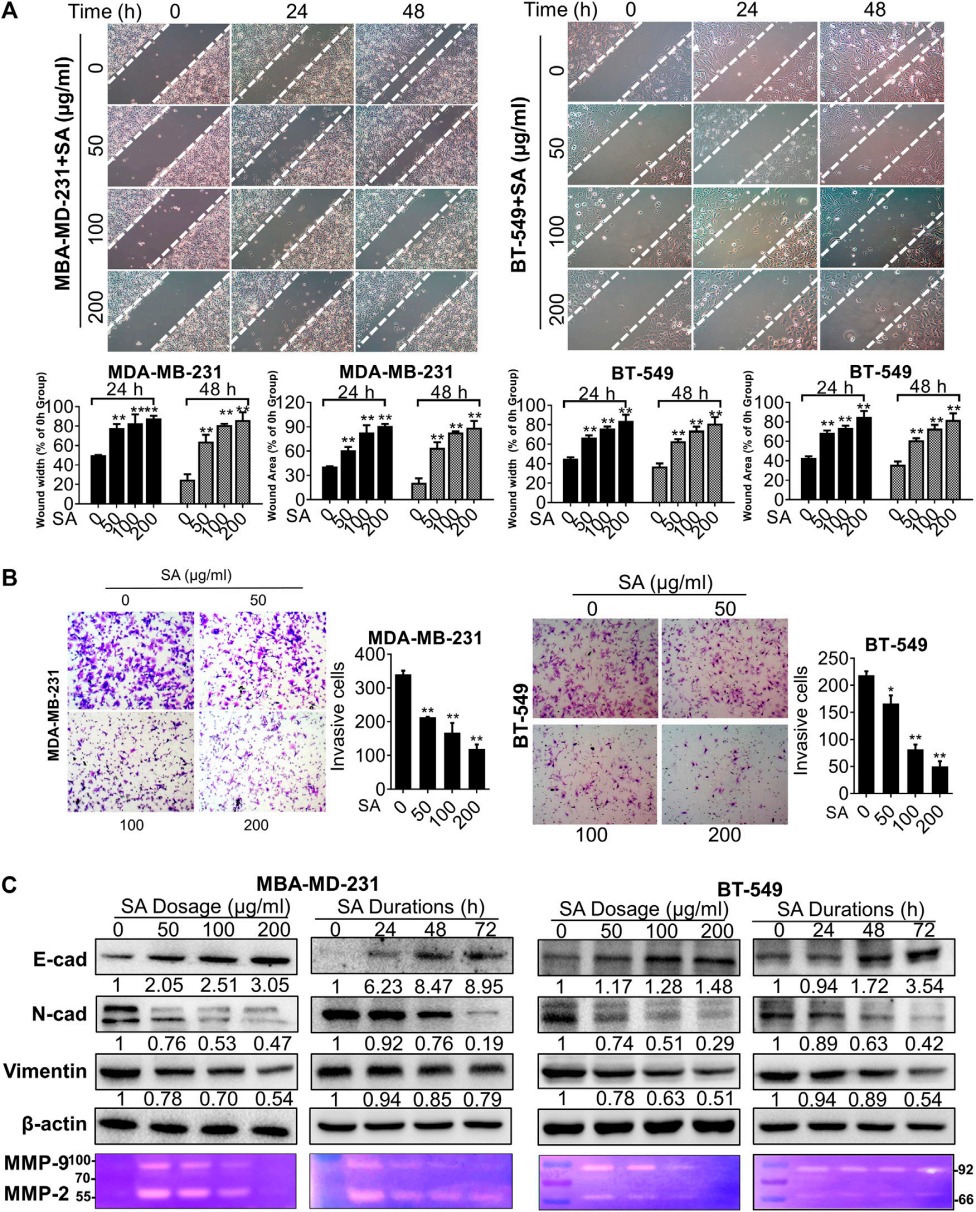
SA suppresses migration and invasion in metastatic cells. **(A)** Representative phase-contrast images for wound healing assays. *Left*: quantification of migration distances; *right*: quantification of migration healing areas. **(B)** Representative images **(*left*)** and quantification **(*right*)** of decreased cell number in transwell chambers with or without SA (0–200 μg/ml). **(C)** Western blotting analysis of E-cad, N-cad and vimentin as well as gelatin zymography detection of MMP-2, and MMP-9 after the indicated SA treatment. All values represent the mean ± SD (*n* = 3, *p < 0.05, **p < 0.01).

Then, we investigated the *in-vitro* influence of SA on the infiltration potential of the high-metastatic breast cancer cell lines, MDA-MB-231 and BT-549 cells. The potential of SA on the migrative ability was assessed by wound healing assays in such breast cancer cells. As indicated in Figure 2A, the gap widths and areas of the untreated group were narrowed more rapidly compared to those of the SA group from 0 to 48 h, implying that SA inhibited the migrative ability of both indicated cells at a dose- and time-dependent manner. Furthermore, chamber invasive assay showed that the invasive cell number was significantly reduced following SA treatment at 50–200 μg/ml, demonstrating that SA administration weakened the invasive potential of breast-cancer cells (Figure 2B). Certain processes, such as the EMT and degradation of the basement membrane, are critical for malignant invasive abilities of cancer cells (Li et al., 2019). In the present study, three EMT-related proteins E-cad, N-cad, and vimentin and two proteolytic enzymes MMP-2 and MMP-9 were selected as targets to investigate the influence of SA in the indicated breast cancer cells. As expected, SA treatment dose- and time-dependently led to decreases in vimentin and N-cadherin as well as an increase in E-cadherin in both cells by western blotting analysis. A gelatin zymography further demonstrated that both the MMP-9 (approximately at 92 kDa) and MMP-2 (approximately at 66 kDa) activities were apparently suppressed by SA, addressing the anti-metastatic capability of SA (Figure 2C). Overall, the above observations demonstrated that SA inhibited metastasis of breast cancer cells *in vitro*.

### Network Pharmacology Analysis of *Sanguisorba Officinalis* L.

**FIGURE 3:**
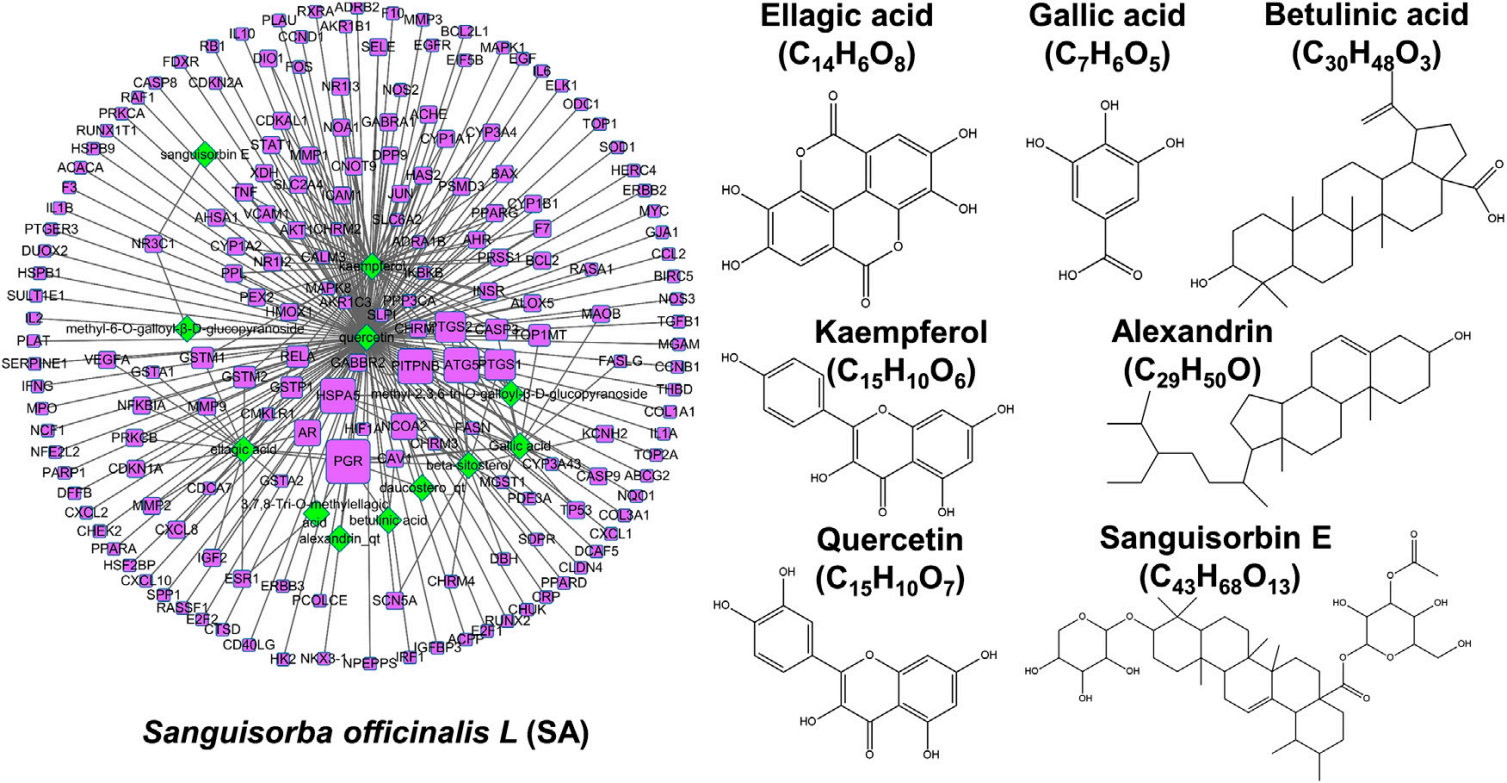
Network pharmacology analysis of SA. ***Left:*** an established ingredient-target network of SA. Ingredients and targets were respectively visualized by green-diamond and purple-square nodes, whose sizes were depending on degree values. ***Right:*** The structures of representative compounds of SA.

The criteria of OB ≥ 30% and DL ≥ 0.1 was set for the purpose of screening active chemicals from SA. In Figure 3, SA produced 12 candidate chemicals as well as 180 potential targets after eliminating duplicates. Furthermore, it was further revealed that there also existed 192 nodes and 283 ingredient-target interactions in this ingredient-target construction of SA. Importantly, many compounds in SA have been proven to be natural anti-cancer agents. In particular, ellagic acid (MOL005858) has been shown to restrain pulmonary metastasis mainly *via* targeting ACTN4 and subsequently destroying *β*-catenin stabilization in breast cancer stem cells (Wang et al., 2017). In addition, betulinic acid suppressed erobic glycolysis in breast cancer *via* Cav-1/NF-κB/c-Myc signaling (Jiao et al., 2019). Quercetin (MOL000098) has been reported to inhibit metastatic potential of human hepatocellular carcinomas by decreasing *p*-Akt, MMP-2, and MMP-9 (Lu et al., 2018). Taken together, our results confirmed that SA may also exert inhibitory effects against highly metastatic cancer diseases.

### Construction of an Ingredient-Target-Breast Cancer Network of *Sanguisorba Officinalis* L.

**FIGURE 4:**
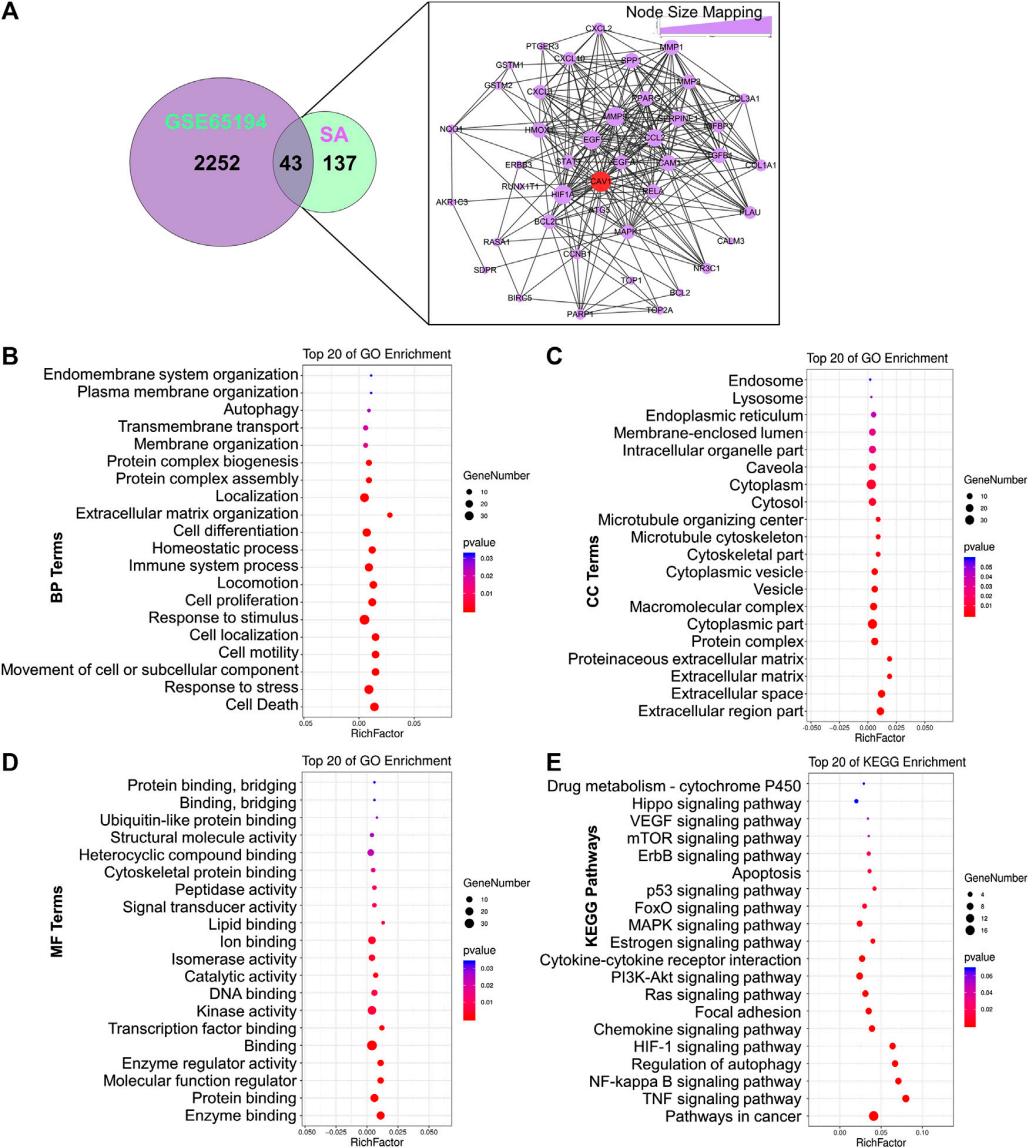
Establishment of an ingredient-target-breast cancer network of SA. **(A)** Among the overlapping DEGs between SA and GSE65194 datasets, CAV-1 was recognized as the hub target due to its largest degree in the node-size mapping. Bubble diagram showed the GO terms of **(B)** biological processes (BPs), **(C)** cellular components (CCs), as well as **(D)** molecular functions (MFs), and **(E)** KEGG terms for enrichment analysis.

To thoroughly reveal the anti-metastatic mechanism of SA, a total of 2,298 DEGs were identified using GSE65194 microarray sets with the criteria of FC ≥ 1.5 as well as *p* ≤ 0.05 by GEO2R analysis. Then, a venn diagram was constructed and displayed 43 common targets between SA and GSE65194, including CAV-1, HIF-1A, EGF, MMP-9, MMP-1, MMP-3, STAT-1, PPARG, CCL-2, BCL2L1, VEGF-A, MAPK-1, CCNB-1, and RELA. According to node-size mapping, CAV-1 was recognized with the largest node size ≥30 on the basis of “degree” among the aforementioned target genes (Figure 4A), implying that Cav-1 might most likely influence the regulatory network of SA against breast cancer.

To identify the functions and mechanisms of SA, we next conducted GO and KEGG pathway-enrichment analysis based on the background of all human genes. For GO-term analysis, biological-process (BP) analysis revealed that cell death (such as autophagy) and cell growth and metastasis (e.g., movement of cells or subcellular components, cell motility, cell localization, cell proliferation, and extracellular matrix organization) were most significantly associated with SA action (Figure 4B); Cellular-component (CC) analysis indicated that the key terms were composed of extracellular regions, extracellular space, the extracellular matrix, and caveola (Figure 4C); Molecular-function (MF) analysis indicated that the top-three enriched terms were enzyme binding, protein binding, and regulation of molecular function (Figure 4D). For KEGG pathway analysis, the anti-cancer mechanisms of SA might be tightly associated with multiple interfered pathways including TNF signaling pathway, NF-κB signaling pathway, regulation of autophagy, Hif-1α signaling, chemokine signaling pathway, focal adhesion signaling, Ras signaling pathway, PI3K-Akt signaling, FoxO signaling, p53 signaling and VEGF signaling (Figure 4E).

### Validation and Identification of *Sanguisorba Officinalis* L. as a Late-Stage Autophagic Inhibitor in Suppressing Breast Cancer Metastasis

**FIGURE 5:**
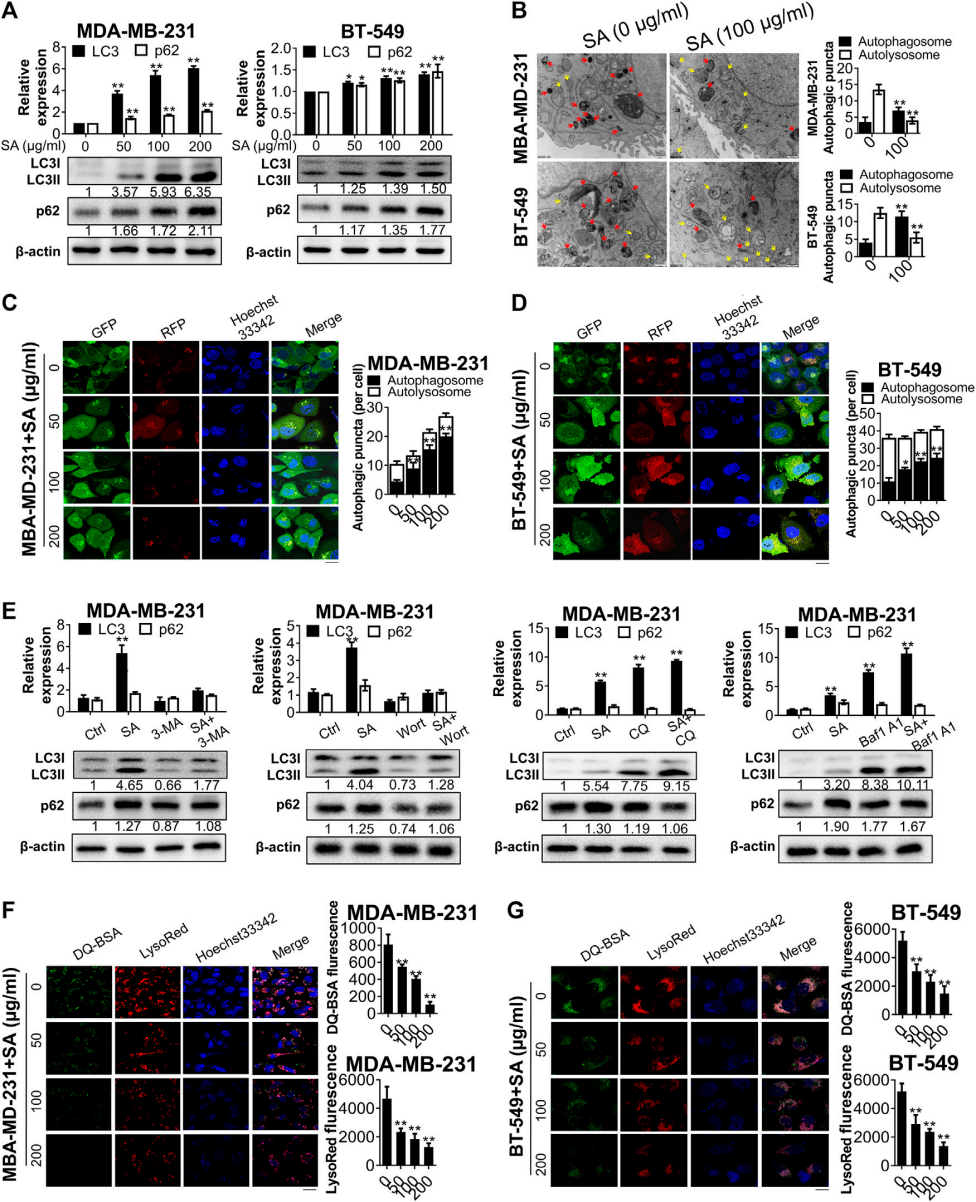
SA inhibits autophagic flux in metastatic cells. **(A)** LC3-II and p62 expressions in MDA-MB-231 and BT-549 cells in exposure to different concentrations (0–200 μg/ml) of SA for 48 h. **(B)** Representative electron micrographs of the cells with SA (100 μg/ml) for 48 h. The yellow arrows represented autophagosomes, whereas the red arrows represented autolysosomes. **(C,D)** Fluorescence photographs of MDA-MB-231 and BT-549 cells transfected with an LC3-GFP-mRFP reporter after SA treatment (0–200 μg/ml) for 48 h. Scale bar: 10 μM. **(E)** Representative bands of LC3-II and p62 in SA-treated MDA-MB-231 cells in addition to 3-MA (10 mM), wortmannin (Wort, 2 μM), CQ (30 μM), or bafilomycin A1 (Baf1, 100 nM) for 48 h. **(F,G)** The fluorescence photographs of SA-treated cells labeled with LysoRed or DQ-BSA. Scale bar: 20 μM. All values represent the mean ± SD (*n* = 3, *p < 0.05, **p < 0.01).

Since both BP and KEGG analysis suggested that autophagy was essential for the anti-cancer function of SA, we next evaluated the effect of SA on autophagic flux in breast cancer cells MDA-MB-231 and BT-549. First, we monitored the protein levels of LC3-II, which is a hallmark of autophagic induction by converting from its cytoplasmic form of LC3-I into the lipidic LC3-II. SA treatment dose-dependently increased the endogenous LC3-I-to-LC3-II conversion in the indicated cells. The expressions of p62/SQSTM1 were also evaluated. Increased p62 reflects a blockade of autophagic flux owing to its constitutive degradation during autophagy. In the present study, SA treatment after 48 h led to dose-dependent increases in p62 expression levels in both cells by western blotting analysis, indicating that SA may exert inhibitory effects on autophagy rather than enhancing autophagic flux (Figure 5A). Furthermore, transmission electron microscopy revealed that SA administration apparently increased the formation of autophagosomes with double membranes (Aps, yellow arrows), while it decreased acidic autolysosomes with single membrane (ALs, red arrows) in comparison with those of control cells in both cell lines (Figure 5B). In addition, we also transfected breast cancer cells with a GFP-mRFP-LC3 reporter in which autophagosomes were indicated by yellow puncta overlapped by green (GFP) and red (mRFP) fluorescent signals, while free red puncta were considered to represent autolysosomes. As shown in Figures 5C and 5D, the numbers of yellow puncta (autophagosomes) were increased following SA treatment in both cancer cells. However, the increased LC3-II expression as well as number of autophagic puncta by SA may be attributable to either increased formation or impaired degradation of autophagosomes (Zhang et al., 2019). To distinguish the two aforementioned possibilities, we administered SA with either early-stage autophagic inhibitors (3-methyladenine [3-MA], 10 mM); or wortmannin, 2 μM) or late-stage autophagic inhibitors (chloroquine [CQ], 30 μM); or bafilomycin A1, 100 nM), and evaluated endogenous LC3-I-to-LC3-II conversion *via* western blotting. The induction of SA on LC3-II accumulation was abrogated by the upstream autophagic inhibitors, 3-MA/wortmannin, while CQ/bafilomycin A1 aggravated LC3-II conversion by SA, suggesting that SA might inhibit the degradation of autophagosomes (Figure 5E). Moreover, both LysoRed and DQ-BSA were dequenched in SA-treated cells, supporting that SA might impair lysosomal function by decreasing intracellular proteolysis in breast cancer cells (Figures 5F,G). Collectively, our data suggest that SA may possibly be a late-stage autophagic inhibitor in high-metastatic breast cancer cells.

**FIGURE 6:**
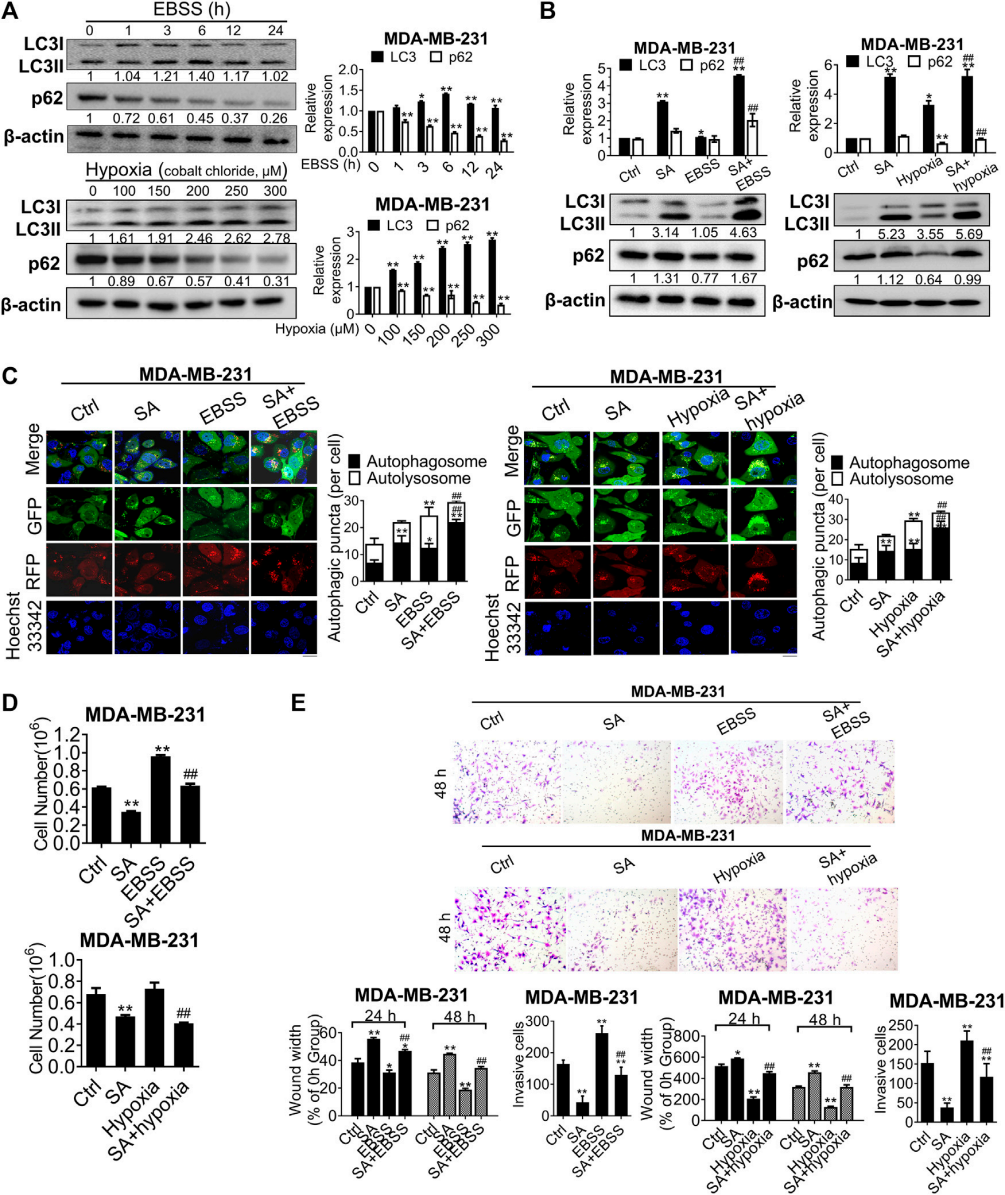
SA suppresses autophagy-mediated metastatic processes during starvation or hypoxia. **(A)** LC3 and p62 expressions in cells co-treated with EBSS or cobalt chloride by western blotting detection. EBSS treatment for 6 h or the hypoxic treatment with cobalt chloride at 200 μM were selected for the following experiments. All values represent the mean ± SD (*n* = 3, *p < 0.05, **p < 0.01). **(B)** LC3 and p62 levels in MDA-MB-231 cells with SA (100 μg/ml) alone or combination with either a 6-h treatment of EBSS **(*Left*)** or cobalt chloride at 200 μM **(*Right*)**. Values represent the mean ± SD (*n* = 3, *p < 0.05, **p < 0.01 *vs.* Ctrl, ##*p* < 0.01 *vs.* EBSS or hypoxia). **(C)** Representative images of autophagic flux in MDA-MB-231 cells with or without SA (100 μg/ml) during starvation or hypoxic conditions by a LC3-GFP-mRFP reporter. Scale bar: 10 μM. Values represent the mean ± SD (*n* = 3, *p < 0.05, **p < 0.01 *vs.* Ctrl, ##*p* < 0.01 *vs.* EBSS or hypoxia). **(D)** Cell counting assays and **(E)** wound healing assays as well as transwell invasion assays reflected the influences of SA on cell growth or invasiveness under starvation (EBSS) or hypoxia (cobalt chloride). Values represent the mean ± SD (*n* = 3, *p < 0.05, **p < 0.01 *vs.* Ctrl, ##*p* < 0.01 *vs.* EBSS or hypoxia).

Autophagy is activated to maintain tumor growth and progression in response to the extremely severe TME, such as that during starvation and hypoxia (Kimmelman and White, 2017). In the present study, this phenomenon was further confirmed by the increased LC3-II and decreased p62 levels in nutrient-free conditions with Earle’s balanced salt solution (EBSS) or hypoxic conditions induced by cobalt chloride (Figure 6A). To further explore whether the metastatic-inhibitory effects of SA were associated with autophagic modulation, we next investigated the interaction effects between SA and the aforementioned autophagic activators. Western blotting revealed that SA promoted a more obvious accumulation of LC3B-II in either nutrient-free or hypoxic conditions, by comparison with that in normal growth conditions (Figure 6B). We also assayed autophagic flux using the mRFP-GFP-LC3B reporter *via* fluorescent microscopy and found that either EBSS or cobalt chloride administration increased both yellow and red puncta, whereas co-incubation of cells with SA and autophagic activators particularly increased yellow puncta in comparison with that of autophagic activators alone or SA alone (Figure 6C). We then analyzed the metastasis-associated capability of MDA-MB-231 cell lines in autophagy-stimulating conditions (starvation and hypoxia) by carrying out cell counting assays, wound healing assays, and transwell invasion assays. It was found that either starvation or hypoxia abolished the anti-cancer effects of SA on proliferation (Figure 6D), migration, and invasion (Figure 6E) *in vitro*. Overall, these findings demonstrate that SA suppressed breast cancer proliferation and metastasis by inhibiting late-stage autophagy.

### *Sanguisorba Officinalis* L. Exerts Anti-metastatic Effects by Suppressing the Hif-1α/Cav-1 Autophagic Axis

**FIGURE 7:**
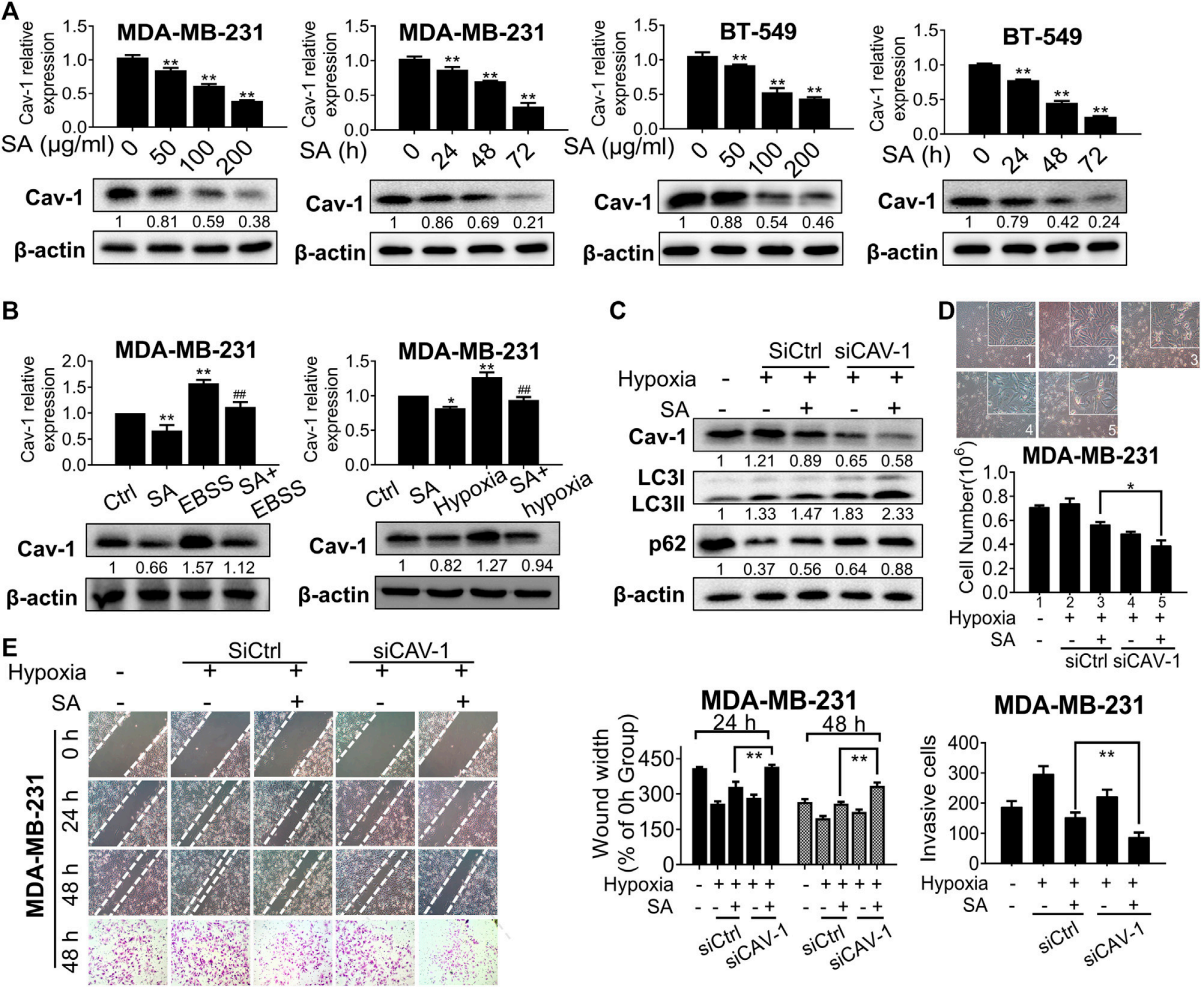
SA inhibits Cav-1 expression and blocks late-phase autophagy and subsequent metastatic processes. **(A)** The expression levels of Cav-1 in MDA-MB-231 and BT-549 cells with different concentrations (0–200 μg/ml) or time intervals (24–72 h) of SA. All values represent the mean ± SD (*n* = 3, *p < 0.05, **p < 0.01). **(B)** The expression levels of Cav-1 in cells treated with SA (100 μg/ml) during starvation or hypoxic conditions. Values represent the mean ± SD (*n* = 3, *p < 0.05, **p < 0.01 *vs.* Ctrl, ##*p* < 0.01 *vs.* EBSS or hypoxia). **(C)** Cav-1, LC3-II, and p62 protein levels in MDA-MB-231 cells transfected with siCAV-1 with or without SA (100 μg/ml) during hypoxia for 48 h. Values represent the mean ± SD (*n* = 3, *p < 0.01 SA + empty vector group during hypoxia *vs.* SA + siCAV-1 group during hypoxia). **(D)** Cell counting assays and **(E)** wound healing assays as well as transwell invasion assays showed that SA suppressed breast-cancer growth and metastasis in a Cav-1-dependent manner under hypoxic stress. Values represent the mean ± SD (*n* = 3, *p < 0.05, *p < 0.01 SA + empty vector group during hypoxia *vs.* SA + CAV-1 group during hypoxia).

Since Cav-1 was found to be the core hub target of SA, we hypothesized that the anti-autophagic and anti-metastatic activities of SA were closely correlated with Cav-1. To test our hypothesis, we firstly evaluated the influence of SA on Cav-1 expression after the indicated treatment. We found that SA inhibited Cav-1 expression levels in a dose- and interval-dependent way for both cancer cells (Figure 7A). We also evaluated the actions of SA on Cav-1 signaling under the aforementioned autophagy-activating chemicals. Cav-1 expression was notably upregulated by either EBSS or hypoxia treatment in comparison with that in the control group, while a combination of SA and autophagy-activating chemicals was associated with a lower Cav-1 expression than that of either EBSS or hypoxia administration alone (Figure 7B). Next, we continued to investigate whether Cav-1 was critical for the anti-autophagic and anti-metastatic activities of SA by decreasing Cav-1 in SA-treated cells under hypoxic conditions. It was found that the stimulating effects of SA on both LC3-II and p62 expression levels were exacerbated following Cav-1 downregulation (Figure 7C). Furthermore, SA was found to efficiently suppress metastatic processes induced by hypoxia. Particularly, cell counting assays, wound healing assays, and transwell invasion assays showed that SA dose-dependently inhibited hypoxia-induced cell proliferation, migration, and invasion. Intriguingly, Cav-1 silencing not only suppressed the migratory-stimulating effect of hypoxia but also enhanced the ability of SA in suppressing metastasis, indicating that Cav-1 may act as the main pharmaceutical target of SA in suppressing breast cancer metastasis under hypoxic stress (Figures 7D,E). Taken together, our data show that SA inhibited Cav-1 levels to block late-phase autophagy and subsequently delay breast-cancer progression under hypoxic stress.

**FIGURE 8:**
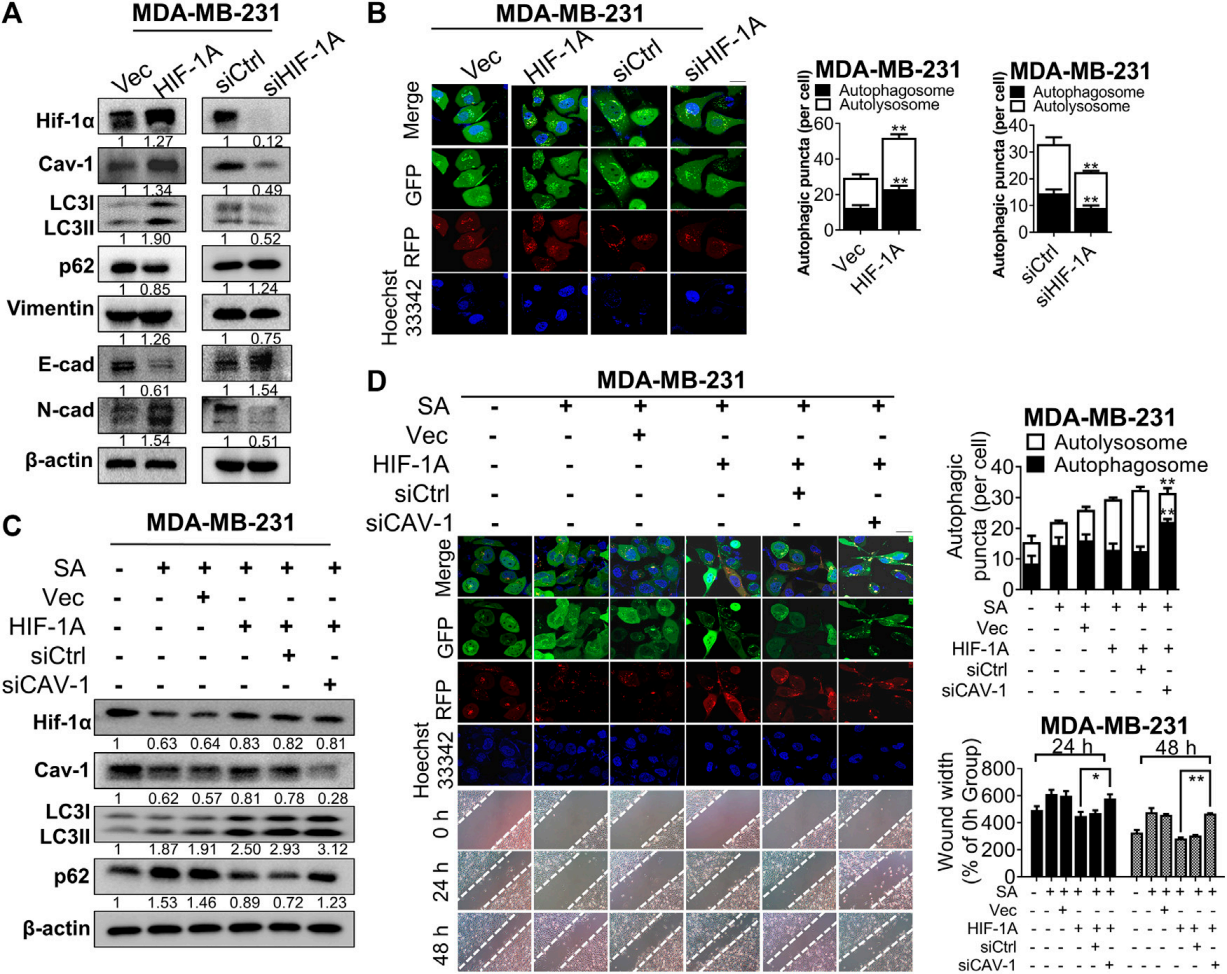
SA exerts anti-metastatic effects partially *via* Cav-1/Hif-1α signaling. **(A)** The levels of Hif-1α, Cav-1, LC3, p62, vimentin, E-cad, and N-cad in MDA-MB-231 cells before or after the transfection of HIF-1A plasmid or siHIF-1A. **(B)** Representative fluorescence photographs showing autophagic activities of MDA-MB-231 cells transfected with or without HIF-1A plasmid or siHIF1-A. Scale bar: 10 μM. All values represent the mean ± SD (*n* = 3, **p < 0.01). **(C)** The levels of Hif-1α, Cav-1, LC3, and p62 in SA-treated MDA-MB-231 cells transfected with HIF-1A plasmid with or without siCAV-1 during hypoxia were measured *via* western blotting analysis. **(D)** Autophagic flux was determined by a LC3-GFP-mRFP reporter and migration distances were detected by wound healing assays in SA-treated MDA-MB-231 cells after the indicated genetic modifications. Scale bar: 10 μM. Values represent the mean ± SD (*n* = 3, **p < 0.01 SA + HIF-1A group *vs.* SA + HIF-1A group + siCAV-1).

Shun et al. (2016) revealed that administration of hypoxia inducer, cobalt chloride, would enhance expression for the hypoxia-mediating factor Hif-1α and subsequent switch on the beginning of autophagy. In our observation, Hif-1α signaling was found to be associated with the anti-cancer mechanisms of SA, as revealed by KEGG pathway-enrichment analysis. Hence, we continued to reveal the interactions between Hif-1α expression and the inhibitory effect of SA on Cav-1 expression, as well as the anti-autophagic/anti-metastatic potential of SA. Exogenous overexpression or silence of Hif-1α resulted in corresponding alternations in Cav-1 expressions, implying a positive regulatory relationship between Hif-1α and Cav-1. In addition, Hif-1α overexpression significantly induced upregulation of various autophagy-related and EMT-associated genes, suggesting that Hif-1α may trigger autophagy and subsequently induce breast cancer metastasis. Consistently, Hif-1α silencing led to absolutely controversial results (Figure 8A). Furthermore, Hif-1α upregulation distinctly enhance autophagic flux for the generation of autophagosomes and/or autolysosomes, whereas Hif-1α silence apparently interfered with autophagosomal formation based on our LC3-mRFP-GFP reporter assay (Figure 8B). To further investigate the regulation of Cav-1 by SA in response to Hif-1α-induced autophagy, we additionally used siRNAs to downregulate Cav-1 in Hif-1α^high^ MDA-MB-231 cells in addition with SA (Figure 8C). Cav-1 downregulation abolished the promoting effects of Hif-1α on autophagic activation. Particularly, Hif-1α-mediated LC3-II overexpression was further upregulated following Cav-1 deficiency, which was accompanied by increased P62 expression (determined by Western blotting). As shown by our LC3-mRFP-GFP reporter, SA increased yellow puncta in MDA-MB-231 compared with that in the untreated group, while Hif-1α upregulation led to increases in both red and green puncta even in the presence of SA. More importantly, the number of Hif-1α-enhanced yellow puncta was further increased following Cav-1 silencing in SA-treated MDA-MB-231 cells (Figure 8D). Our data also showed that Cav-1 deficiency led to ameliorated metastasis that was enhanced by Hif-1α, as indicated by wound healing assays (Figure 8D). Taken together, our data suggest that SA exerted anti-metastatic effects, at least partly *via* the Hif-1α/Cav-1 autophagic axis.

### *Sanguisorba Officinalis* L. Suppresses Growth and Metastasis *in vivo* and *ex-vivo* Breast Cancer Xenotransplantation Model

**FIGURE 9:**
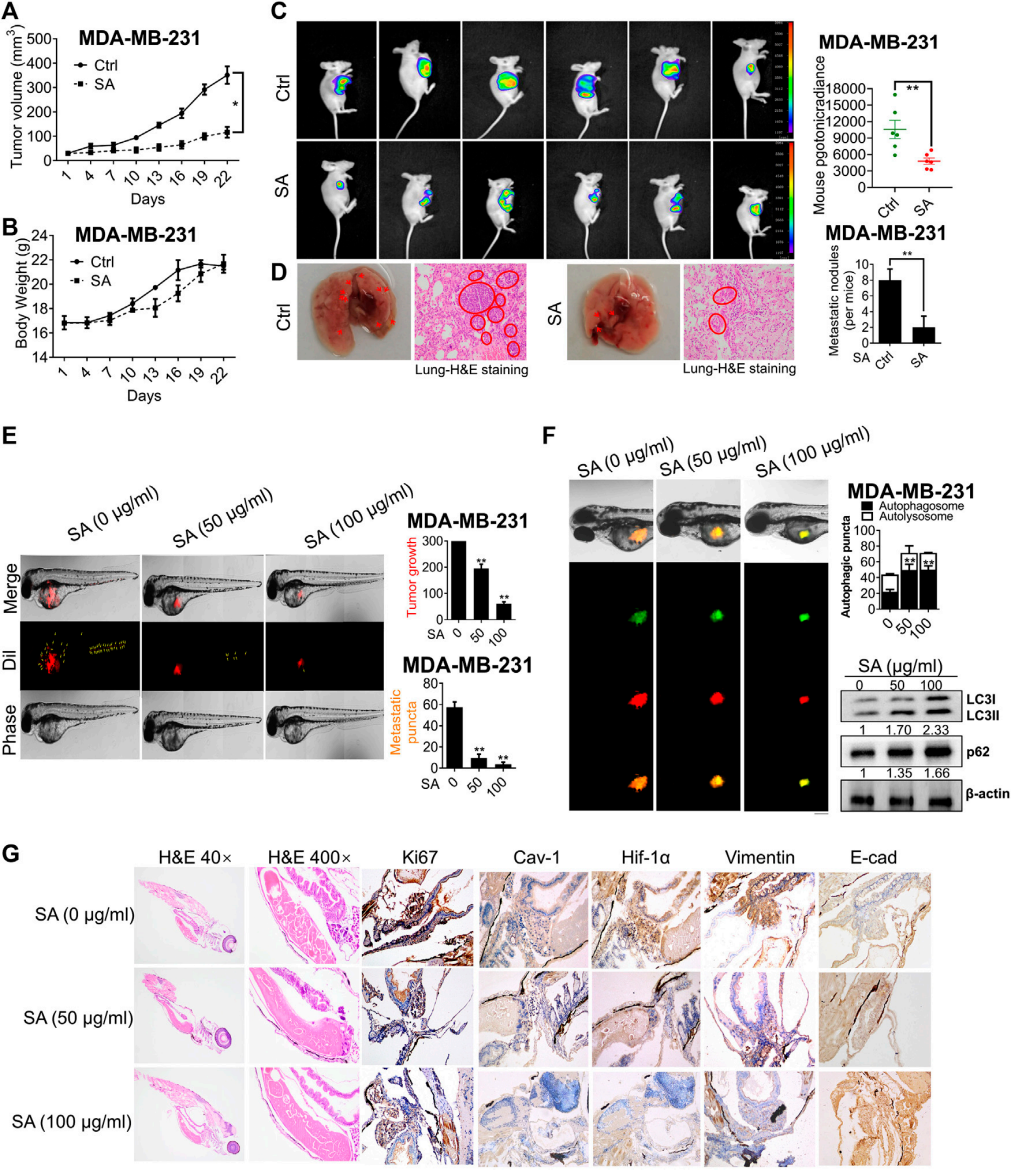
SA suppresses breast cancer growth and metastasis *in vivo* and *ex vivo*. **(A)** Tumor volume, **(B)** body weight, **(C)** tumor-bioluminescence imaging, and **(D)** pulmonary metastatic nodules in nude mice bearing MDA-MB-231 cells with or without SA administration (*n* = 6, *p < 0.05, **p < 0.01). **(E)** The tumor foci in the control and SA-treated zebrafishes. Red fluorescence represented Dil-stained MDA-MB-231 cells, and yellow arrow labeled the position of disseminated tumor. **(F)** The effects of SA on autophagic flux was measured by LC3-GFP-mRFP reporter and the autophagy-associated indexes, LC3-II and p62, by western blotting analysis. Scale bar: 50 μM. **(G)** H&E staining and IHC detection of Ki-67, Cav-1, Hif-1α, vimentin, and E-cad expressions for each indicated group (*n* = 3, *p < 0.05, **p < 0.01).

We next investigated the anti-growth and anti-metastasis potential of SA on nude mice. Firstly, MDA-MB-231 cells were subcutaneously injected into the mammary glands of mice models for the establishment of nude mice xenografts. SA exerted a significant inhibition of tumor volume (Figure 9A), while induced little weight loss throughout the entire experiment (Figure 9B). This result was consistent with *in-vivo* bioluminescent imaging, showing that tumor growth was significantly suppressed after SA administration (Figure 9C). To additionally examine the effects of SA on breast cancer metastasis, we established mice model with lung metastasis by tail vein injection. As shown in Figure 9D, fewer metastatic nodules were present in the lung parenchyma of SA-treated group per mice in comparison with control group. In the histological observation by H&E staining, untreated-lung tissues showed abnormal appearances characterized by cancerous tissues, whereas SA administration did not lead to obvious changes in lung histopathology.

To further monitor the effect of SA on breast-cancer metastasis, a red fluorescent dye, Dil, was employed to predict the distribution and progression of MDA-MB-231 cells on zebrafish xenotransplantation model. As presented in Figure 9E, the intensity of red fluorescence in the control group was much brighter than the group with SA. Additionally, the untreated cells ran faster and spread more widely to the distant tail within zebrafish, while SA treatment significantly led to a decreased number of disseminated tumor foci. To further assess the autophagic regulation of SA *ex vivo*, we developed a zebrafish autophagy model by injecting MDA-MB-231 cells stably expressing GFP-mRFP-LC3. As demonstrated, GFP-mRFP-LC3-stained cells that were previously exposed to SA clearly displayed more yellow autophagic vesicles within the body of zebrafish than in the untreated group. What’s more, western blotting further confirmed that SA administration increased both LC3-II and p62 expressions in tumor sections (Figure 9F). This finding was further confirmed by H&E staining, revealing that SA treatment led to increased apoptosis in tumor sections, accompanied by decreased Cav-1, Hif-1α, and vimentin, as well as increased levels of E-cad (Figure 9G). In conclusion, our data suggest that SA exerted anti-cancer and anti-metastatic activities in multiple model systems.

## Discussion

Network pharmacology combined with bioinformatic analysis has deciphered the intricate correlations between TCM formulas and the involved targets and diseases (Hopkins, 2008; Eichler, 2012). Ren et al. (2020) demonstrated that lysine degradation was tightly associated with a protective role and detoxificating function of a TCM formulation of *Yunnan Baiyao*, as demonstrated by a combination of analyses involving network pharmacology and metabolomics. Zhang et al. (2020) revealed a total of 13 potential targets might be associated with the function of *Yinzhihuang* granules against hepatitis B based on network pharmacology, among which TP53, CDK2, CDK6, and BRCA1 might be the core targets of such granules in hepatitis-B treatment by the following molecular-docking verification. Herein, our present study identified 12 active chemicals in SA interacting with 43 genes involved in invasive breast cancer, and Cav-1 was recognized as the core target according to its “degree” in our node-size mapping. Among the core compounds of SA, ellagic acid, gallic acid, betulinic acid as well as quercetin were found to have a close association with the target Cav-1. It was also revealed that ellagic acid and gallic acid as potential anti-cancer candidates of SA based on the bioactivity-guided identification (Wang et al., 2012b). Furthermore, we currently conduct the surface plasmon resonance (SPR) analysis of Cav-1 interacting with the aforementioned chemicals, implying that gallic acid is the potential candidate in SA owing to its strong interaction and competitive inhibition with Cav-1 (data unpublished). Thus, we will continue to explore the anti-cancer as well as autophagic inhibition effects of gallic acid on breast cancer in the future.

Cav-1 is a caveola-forming membranous protein comprised of 178 amino acids, and plays a dual role as a promoter and a suppressor depending on varying cancer types and stages. Particularly, this gene functions predominantly as a tumor suppressor as early as the onset of tumorigenesis, whereas a positive correlation was found between a higher Cav-1 expression with a more advanced level of tumor malignancy and also a worse clinical outcome (Ketteler and Klein, 2018; Qian et al., 2019). Kim *et al* revealed that Cav-1 promoted brain metastasis possibly by regulation of the EMT marker Snail in lung cancer (Kim et al., 2019). Its metastasis-promoting function might be also activated by the small GTPases, Rac-1 and Rab-5 in cancer cells (Diaz et al., 2014). In the present study, we further demonstrated that Cav-1 might act as the main pharmaceutical target of SA in suppressing metastasis on triple-negative breast cancer cell lines. This finding was also consistent with our previous study demonstrating that Cav-1 had higher expression in triple-negative breast cancer cell line MDA-MB-231 compared to other subtype cell lines (Wang et al., 2020). Our pilot study also demonstrated that Cav-1 acts as the upstream regulator of NF-κB in breast cancer (Jiao et al., 2019). Meanwhile, Cav-1 is generally considered as the molecular hub integrating multiple signaling tyrosine kinases, MAPK cascades, EGFR families, as well as NF-κB (Wang et al., 2011; Ma et al., 2016; Kim and Hirabayashi, 2018). Given that Cav-1 reaches the largest node size compared to NF-κB and other targets, it is rationally to select Cav-1 as the main target to study. In our current study, we also found that both MMP-2 and MMP-9 expression were significantly inhibited following SA administration. MMP-2 and MMP-9 comprise the gelatinase family that possesses three fibronectin repeats allowing for extracellular matrix remodeling. MMP-2 is located on chromosome 16 and codes for gelatinase A, and its substrates include gelatin, collagen V, and collagen VI. By contrast, MMP-9 gene is located on chromosome 20 and encodes the gelatinase B protein, and the main substrates for this enzyme include gelatin, collagen IV, and V (Rodríguez et al., 2010). With regard to expression regulation, MMP-2 is usually regulated by tumor necrosis factor-α under the influence of NF-κB transcription factor (Green et al., 2011), while redox-regulated p38 phosphorylation and subsequent AP-1 activation appear to be critical for MMP-9 expression, at least in murine macrophages (Woo et al., 2004). In our current study, network pharmacology analysis indicated that both NF-κB and p38 were involved in the pharmacology action of SA. More importantly, a number of studies implied that Cav-1 acts as the upstream regulator of NF-κB and p38. Therefore, the inhibition effects of SA on Cav-1 might be the upstream factor resulting in the activity reduction of MMP-2 and MMP-9.

Tumor metastasis is responsible for cancer-associated deaths, and effective approaches are still rare. Revealing the underlying mechanisms is essential for developing effective means and agents in cancer metastasis. Herein, both GO and KEGG enrichment suggested that regulation of autophagy was the most significant signaling pathway involved in the actions of SA. Autophagy serves as a core reason for tumor growth and invasiveness. Currently, inhibitors targeting autophagy have been roughly classified into two categories based on specific stages. One class specifically suppresses the induction of autophagy and includes 3-MA, wortmannin, SBI-0206965, UAMC-2526, and NSC185058. In contrast, the other category includes CQ, HCQ, bafilomycin A1, SAR405, ROC-325, Lys05, and DQ661, and this class targets late-phase autophagy *via* either inhibiting fusion of autophagosomes/autolysosomes or suppressing degradation of autolysosomes (Limpert et al., 2018; Zhang et al., 2019). However, the commonly used HCQ and CQ have exhibited disappointing efficacies and off-target toxicities in clinical trials, highlighting alternative requirements for potent and safe agents that regulate autophagy (Long and McWilliams, 2019). In this study, the anti-metastatic capability of SA was found to be tightly associated with its stage-specific inhibition of autophagy. Particularly, SA-treated breast-cancer cells exhibited restriction of cell proliferation, impaired colony formation, and ameliorated metastatic potency in breast cancer cells. Furthermore, western blotting, transmission electron microscopy, and GFP-mRFP-LC3 immunofluorescence identified SA as a potent late-stage autophagic inhibitor by increasing LC3-II conversion, as well as by decreasing acidic vesicular-organelle formation. We further confirmed that SA may inhibit the degradation of autophagosomes by evaluating the synergistic effects of SA with either upstream autophagic inhibitors (3-MA/wortmannin) or downstream autophagic inhibitors (CQ/bafilomycin A1). Additionally, the intensities of both LysoRed and DQ-BSA, representing lysosomal activity, were sharply decreased following exposure to SA. Moreover, our *in vitro* findings were also confirmed by the zebrafish model bearing MDA-MB-231 cells *in vivo*. SA treatment not only significantly led to a decreased number of disseminated tumor foci, but also resulted in decreased autophagic activities at the late stage. Taken together, our findings demonstrated that SA exerted anti-metastatic effects, possibly by regulating autophagy; the suppressive effect of SA on autophagy may be largely due to its induction of lysosomal dysfunction, resulting in failure of engulfed cargo degradation. Interestingly, SA functions similarly to that of CQ-class agents targeting late-phase autophagy, and confers a advantage battling with invasive breast cancer in clinic applications. In comparison with the features of other targeted agents such as CQ, SA exhibits obvious advantages in terms of fewer adverse effects when treating multi-factorial diseases such as cancer.

Autophagy is paradoxically implicated in both cancer-suppressive and -promoting biological activities during metastasis depending on different contextual demands of tumor cells (Galluzzi et al., 2015). In our present study, enrichment analysis indicated that the anti-metastatic role of SA was strongly associated with regulation of autophagy and responses to stressors. Within tumors, autophagy facilitates cancer cells to survive under multiple internal and external stresses including hypoxia, nutrient deficiency, oxidized/aggregated proteins, growth-factor withdrawal, intracellular calcium accumulation, ROS production, ammonia generation, and acidity (Maes et al., 2013; Nagelkerke et al., 2015). The complex interaction between hypoxia (accompanied by the corresponding increase in Hif-1α expression) and autophagic activation has been well studied, particularly in the context of cancer progression. Hif-1α is capable of regulating autophagy by directly targeting autophagic components, including BCL2 and adenovirus E1B 19 kDa-interacting protein 3 (BNIP3), BNIP3-like (BNIP3L)/NIX, beclin 1, phosphatidylinositol three kinase catalytic subunit type 3 (PIK3C3), ATG5, ATG7, and ATG9A. Additionally, Hif-1α also plays a role in autophagy-associated glucose metabolism by regulating lactate dehydrogenase (LDHA), phosphoglycerate kinase 1 (PGK1), glucose transporters 1/3 (GLUT1/3), enolase 1 (ENO1), hexokinases (HK1/2), pyruvate dehydrogenase kinase 1 (PDK1), 6-phosphofructo-2-kinase/fructose-2, and 6-bisphosphatase 3 (PFKFB3) (Wu et al., 2015). In this study, the interaction between Hif-1α and Cav-1 was investigated during autophagy-mediated metastasis in breast cancer. We found that Cav-1 downregulation not only abolished the promoting effects of Hif-1α on autophagic activation, but also led to an ameliorated metastatic capability that was enhanced by Hif-1α in the presence of SA. Furthermore, in the study of Varela-Guruceaga et al. (2018), a hypoxia response elements (HRE) (5′-[AG]CGTG-30) was identified for Hif-1α binding with Cav-1 within its mouse promoter at −442 bp upstream of the transcription starting site (TSS). Furthermore, a study by Wang et al. (2012a) discovered that hypoxia-inducible factor-dependent upregulation of Cav-1 led to increased oncogenic potential for invasive potential in cancer. Therefore, in our next study, we plan to investigate whether Cav-1 transcription can be directly regulated by Hif-1α in human breast cancer, and whether SA can interrupt this interaction.

## Conclusion

In conclusion, our study revealed that SA suppressed triple-negative breast cancer metastasis *via* blocking late-phase autophagy and highlight a novel role of Cav-1 in autophagic control *via* suppressing autolysosomal formation (Supplementary Figure S2).

## Data Availability

The raw data supporting the conclusions of this article will be made available by the authors, without undue reservation, to any qualified researcher.
