# Corrigendum: *Sanguisorba officinalis* L. Suppresses Triple-Negative Breast Cancer Metastasis by Inhibiting Late-Phase Autophagy via Hif-1α/Caveolin-1 Signaling

**DOI:** 10.3389/fphar.2021.810621

**Published:** 2022-01-06

**Authors:** Neng Wang, Gulizeba Muhetaer, Xiaotong Zhang, Bowen Yang, Caiwei Wang, Yu Zhang, Xuan Wang, Juping Zhang, Shengqi Wang, Yifeng Zheng, Fengxue Zhang, Zhiyu Wang

**Affiliations:** ^1^ The Research Center for Integrative Medicine, School of Basic Medical Sciences, Guangzhou University of Chinese Medicine, Guangzhou, China; ^2^ Integrative Research Laboratory of Breast Cancer, The Second Clinical College, Guangzhou University of Chinese Medicine, Guangzhou, China; ^3^ Guangdong Provincial Key Laboratory of Clinical Research on Traditional Chinese Medicine Syndrome, Guangdong Provincial Academy of Chinese Medical Sciences, Guangdong Provincial Hospital of Chinese Medicine, Guangzhou, China

**Keywords:** breast-cancer metastasis, *Sanguisorba officinalis* L., late-phase autophagic regulation, Cav-1, HIF-1α

In the original article, there were mistakes in Figures 2B, 6E as published. In Figure 2B, the invasion image of 100 μg/ml SA in MDA-MB-231 panel was mistakenly uploaded. In Figure 6E, certain invasion images were unintentionally misused during picture assembly and image processing. The corrected Figures 2B, 6E appear below.

**FIGURE 2:**
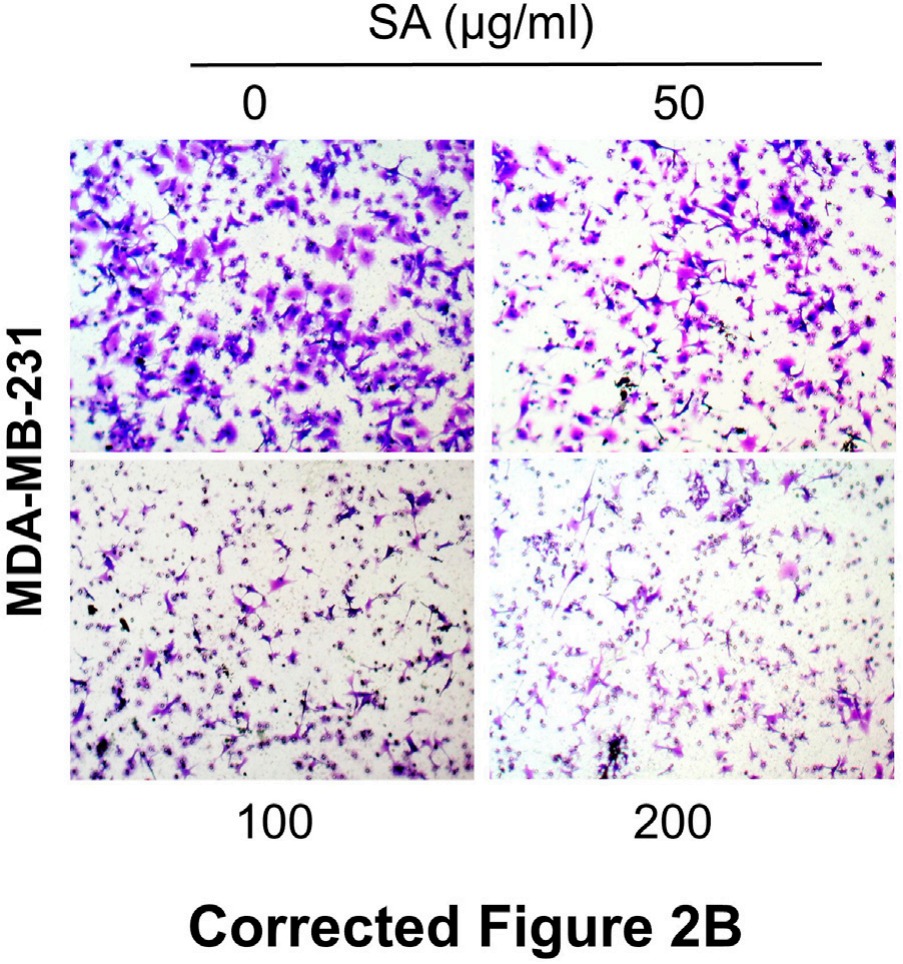
SA suppresses migration and invasion in metastatic cells. **(B)** Representative images of decreased cell number in transwell chambers with or without SA (0–200 μg/ml).

**FIGURE 6:**
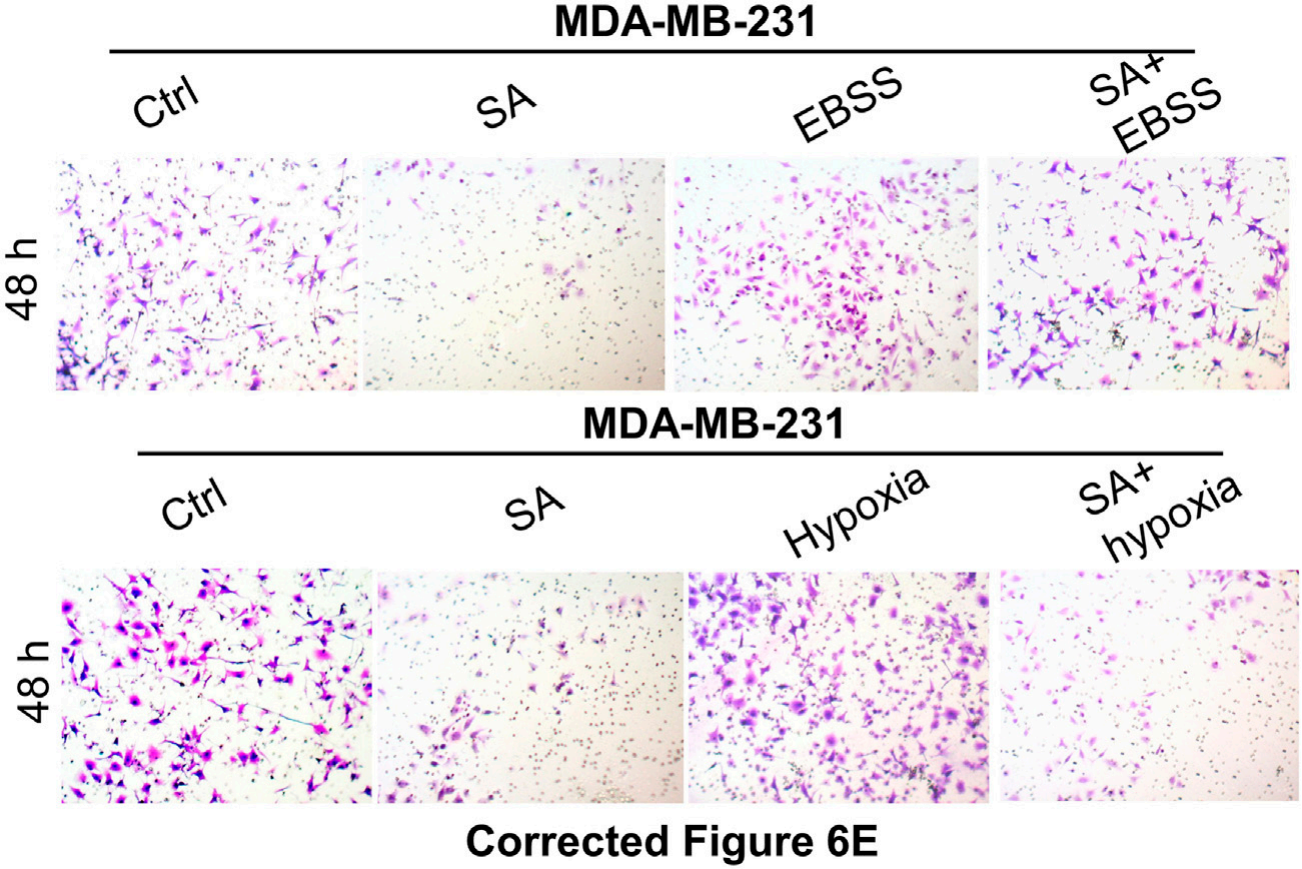
SA suppresses autophagy-mediated metastatic processes during starvation or hypoxia. **(E)** wound healing assays as well as transwell invasion assays reflected the influences of SA on cell growth or invasiveness under starvation (EBSS) or hypoxia (cobalt chloride).

The authors apologize for this error and state that this does not change the scientific conclusions of the article in any way. The original article has been updated.

