# Supplementary figures and images for: Sanguisorba officinalis L. Suppresses Triple-Negative Breast Cancer Metastasis by Inhibiting Late-Phase Autophagy via Hif-1α/Caveolin-1 Signaling
Source: Front Pharmacol. 2020 Dec 14;11:591400. doi: 10.3389/fphar.2020.591400 (PMC7768086; doi:10.3389/fphar.2020.591400)

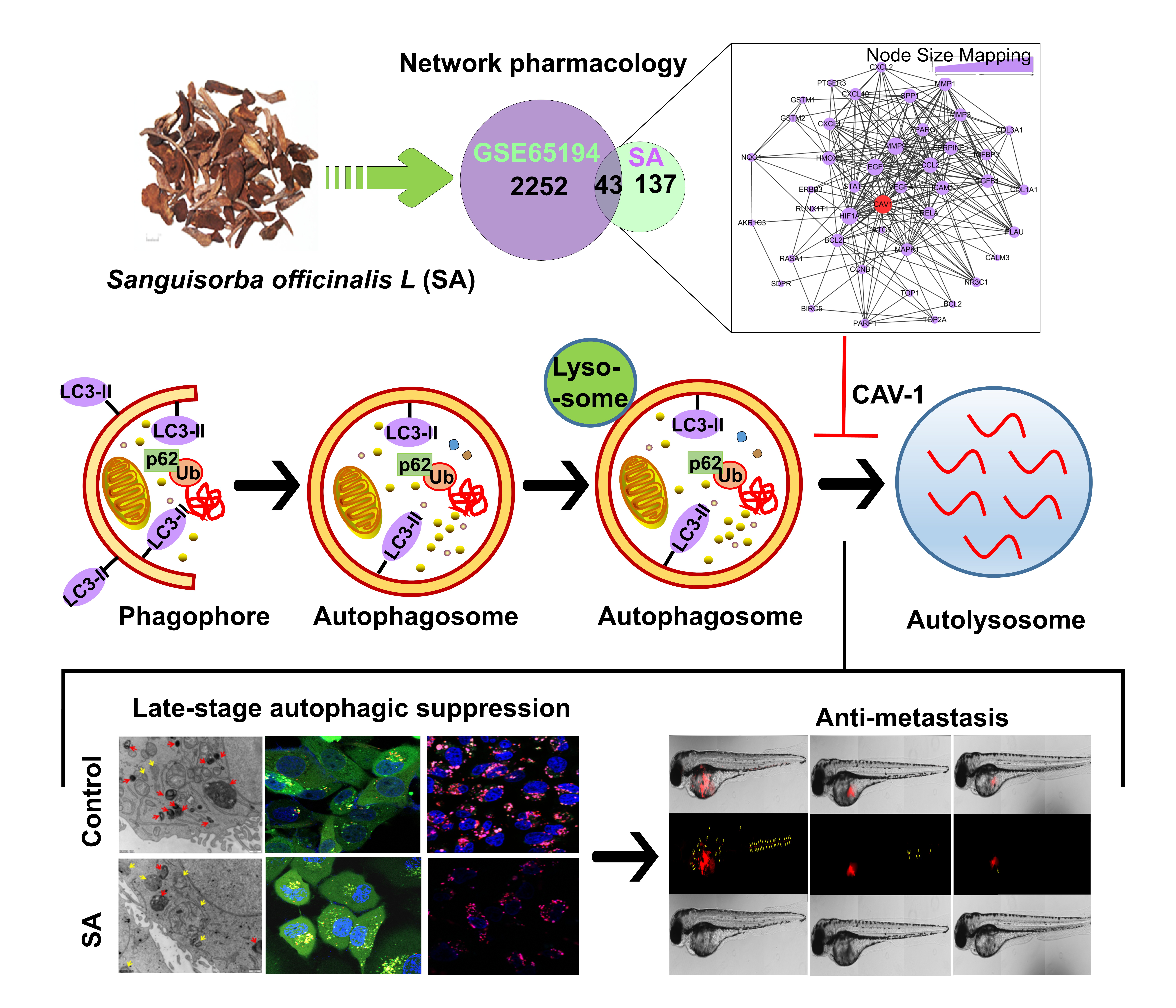

Supplement: Supplementary file 1 [file image2.jpeg]

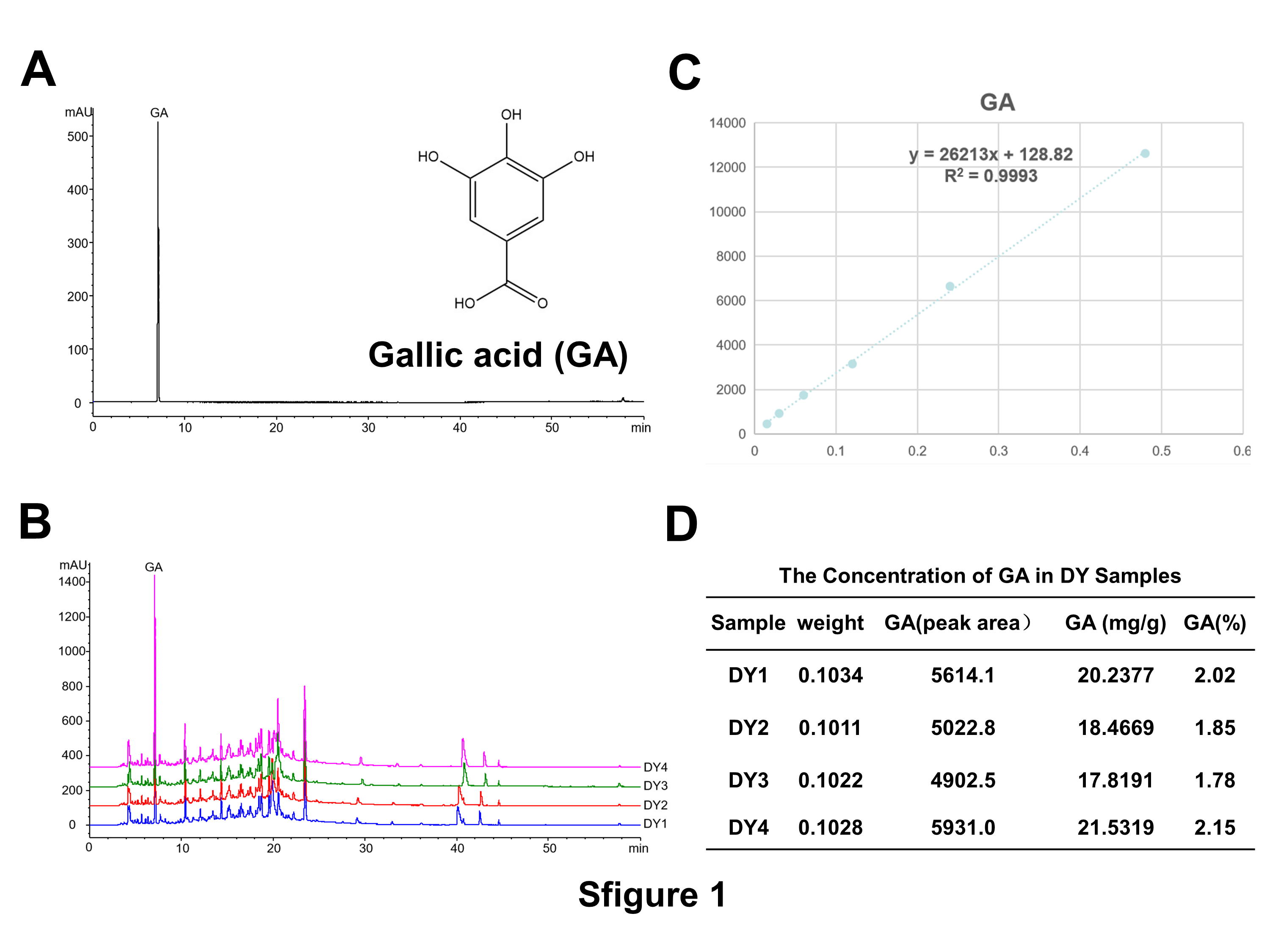

Supplement: Supplementary file 2 [file image1.tif]

Figure 2C

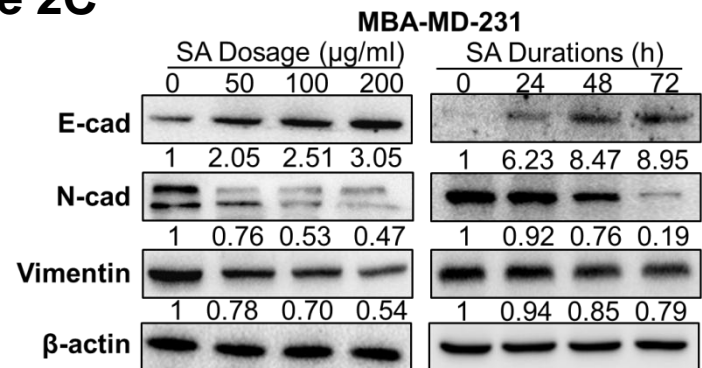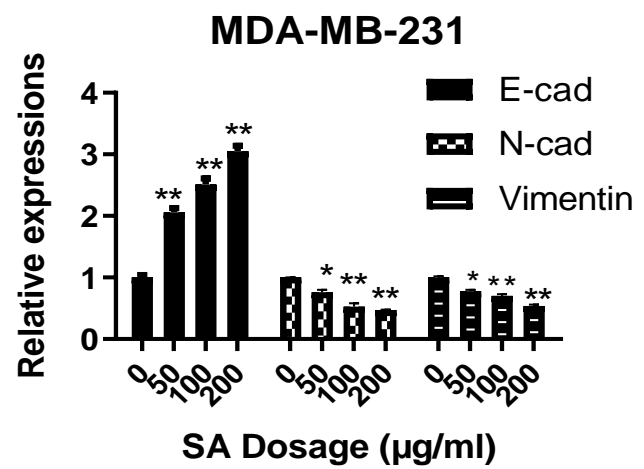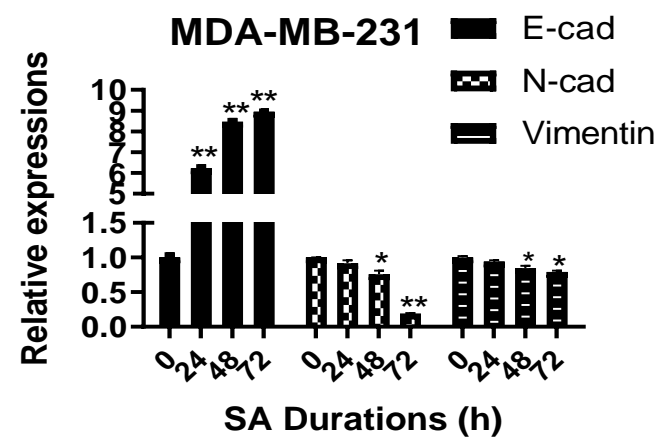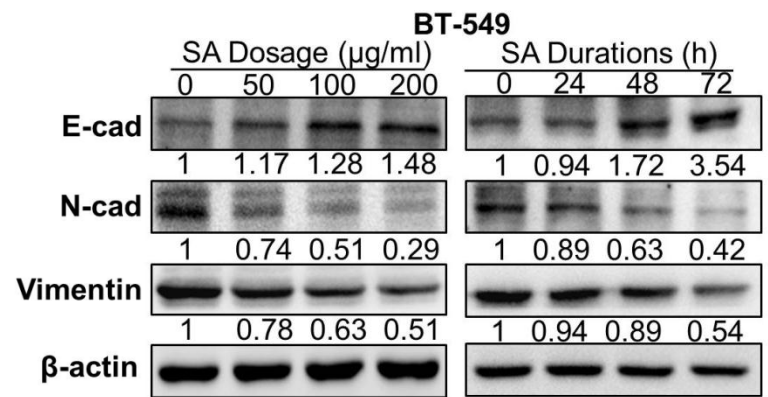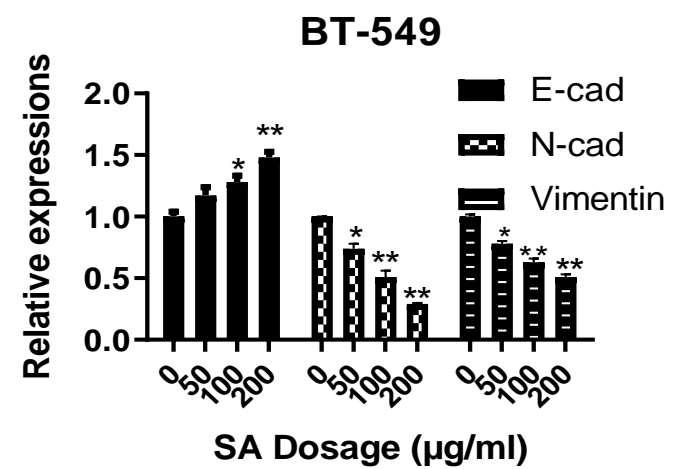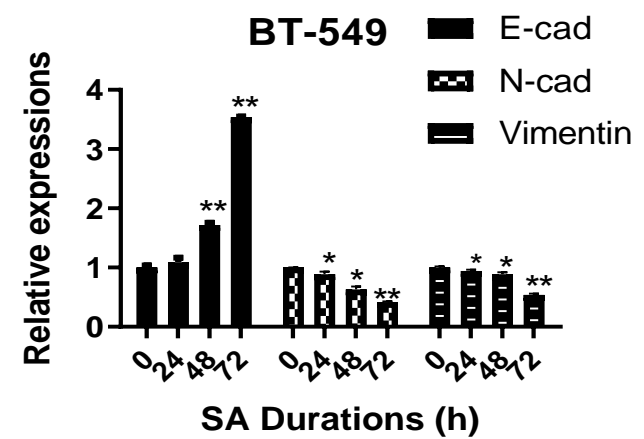

Figure 7C

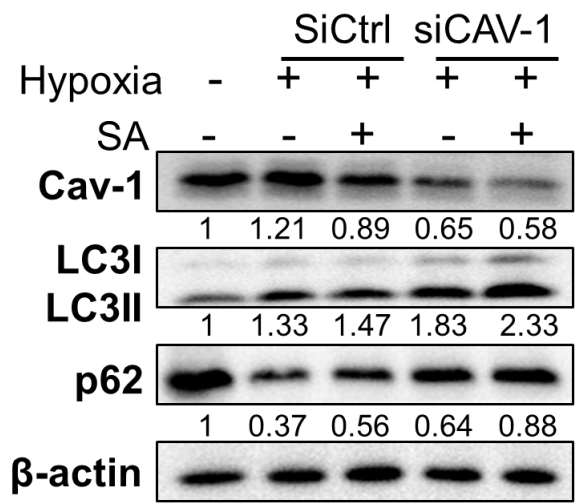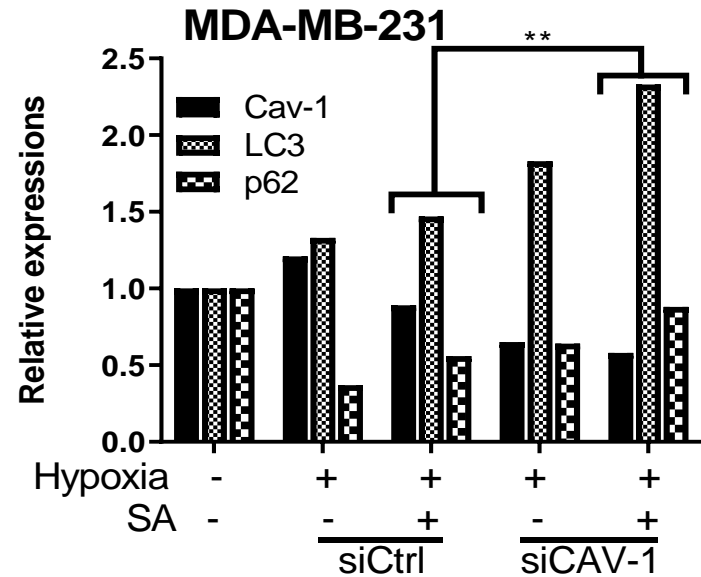

**Figure 8A**

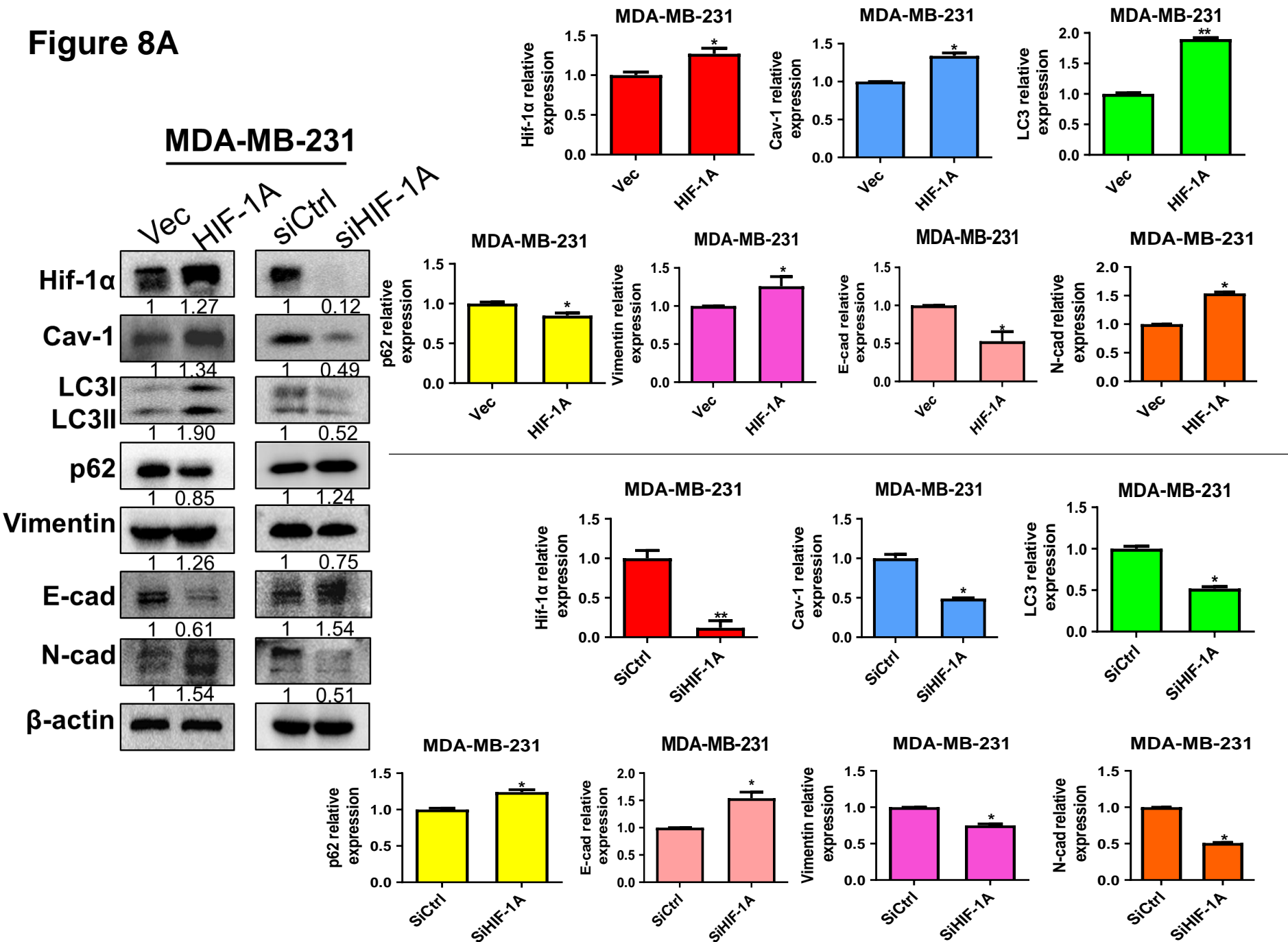

**Figure 8C**

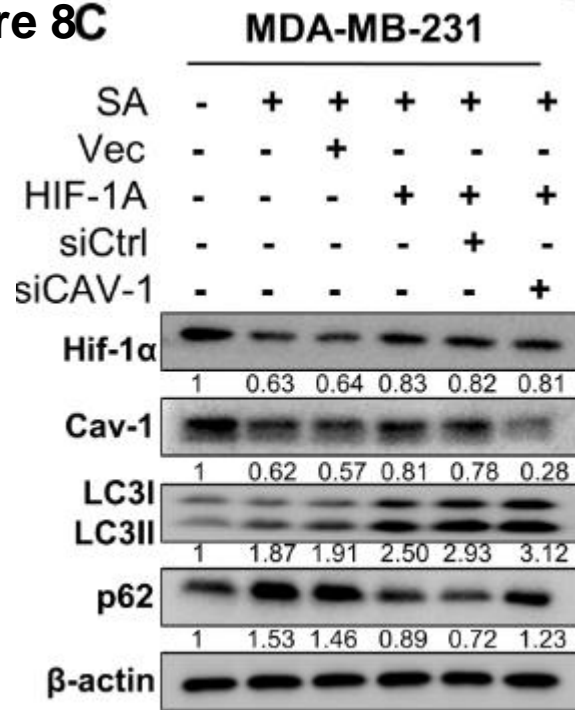

**MDA-MB-231**

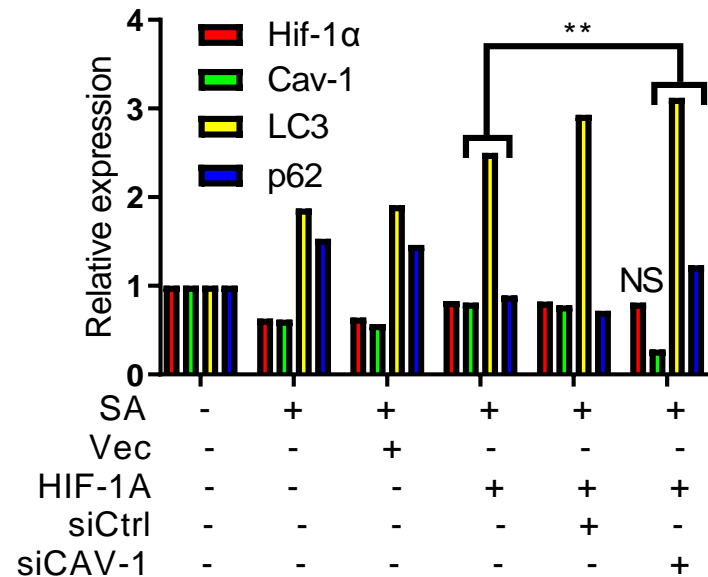

**Figure 9E**

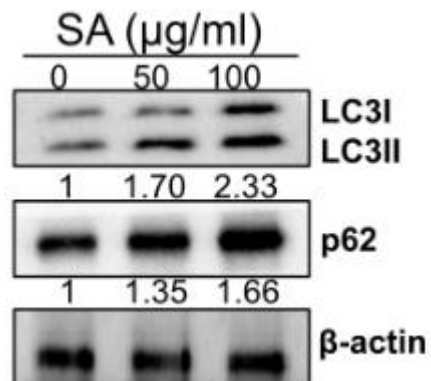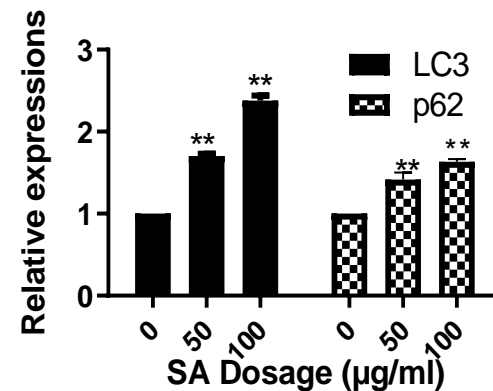

Supplement: Supplementary file 3 [file datasheet1.pdf]
